# Decreased progenitor TCF1 + T-cells correlate with COVID-19 disease severity

**DOI:** 10.1038/s42003-024-05922-2

**Published:** 2024-05-03

**Authors:** Thai Hien Tu, Ami Grunbaum, François Santinon, Alexandra Kazanova, Nicholas Rozza, Richard Kremer, Catalin Mihalcioiu, Christopher E. Rudd

**Affiliations:** 1 Départment of Medicine, Universite de Montreal, Montreal, QC H3T 1J4 Canada; 2 Département de microbiologie, infectiologie et immunologie, Université de Montréal, Montréal, QC H3T 1J4 Canada; 3 Division of Immunology-Oncology, Centre de recherche de l’Hôpital Maisonneuve-Rosemont, Montréal, QC H1T 2M4 Canada; 4 Division of Experimental Medicine, McGill University, Montreal, QC H3A 0G4 Canada; 5 Department of Medicine, Research Institute of the McGill University Health Center, Montreal, H3A 0G4 Canada; 6 Division of Medical Biochemistry, McGill University Health Centre, Montréal, QC Canada; 7 Department of Medical Oncology, McGill University Health Center, Montreal, Quebec Canada

**Keywords:** Diagnostic markers, Infectious diseases

## Abstract

COVID-19, caused by SARS-CoV-2, can lead to a severe inflammatory disease characterized by significant lymphopenia. However, the underlying cause for the depletion of T-cells in COVID-19 patients remains incompletely understood. In this study, we assessed the presence of different T-cell subsets in the progression of COVID-19 from mild to severe disease, with a focus on TCF1 expressing progenitor T-cells that are needed to replenish peripheral T-cells during infection. Our results showed a preferential decline in TCF1+ progenitor CD4 and CD8+ T-cells with disease severity. This decline was seen in various TCF1+ subsets including naive, memory and effector-memory cells, and surprisingly, was accompanied by a loss in cell division as seen by a marked decline in Ki67 expression. In addition, TCF1+ T-cells showed a reduction in pro-survival regulator, BcL2, and the appearance of a new population of TCF1 negative caspase-3 expressing cells in peripheral blood from patients with severe disease. The decline in TCF1+ T-cells was also seen in a subgroup of severe patients with vitamin D deficiency. Lastly, we found that sera from severe patients inhibited TCF1 transcription ex vivo which was attenuated by a blocking antibody against the cytokine, interleukin-12 (IL12). Collectively, our findings underscore the potential significance of TCF1+ progenitor T-cells in accounting for the loss of immunity in severe COVID-19 and outline an array of markers that could be used to identify disease progression.

## Abstract (web summary)

This study shows that severity of disease in COVID-19 patients is characterized by the preferential loss of TCF1+ progenitor T-cells needed to self-renew and replenish the immune system.

## Introduction

The clinical severity of COVID-19 as mediated by the severe acute respiratory syndrome coronavirus 2 (SARS-CoV-2) ranges from asymptomatic to mild self-limiting disease to severe disease manifestations such as acute respiratory distress syndrome (ARDS), neurological symptoms and death^1–3^. It is well established that the virus gains entry to host cells via SARS-CoV-2 spike (S) protein binding to the ACE2 receptors on the cell surface^4–6^. However, efforts to uncover the cause(s) of poor disease outcomes have had only limited success^7–10^. The profound impact of the disease course on the immune system^8,9,11^ and the inflammatory response has been well documented^4,5,12^. Mild cases are associated with antibody and T-cell responses, while severe cases involve the loss of T-cells and reduced antibody responses^5,12–17^. Severe infections are also associated with increased pro-inflammatory cytokines and chemokines including IL6, IL10 and granulocyte-macrophage colony-stimulating factor (GM-CSF)^18^, high neutrophil levels^7,18^, T-cell exhaustion^8^ and decreases in the numbers of regulatory T-cells (Treg)^8,9,16,19–22^. Severe disease also involves the development of severe lymphopenia that affects CD4 + , CD8 + T cells, B cells and natural killer cells^9,10,20,22^. suggest a preferential impact on CD8 + T cells^17,23,24^. Despite this, a clear notion of how activation events are linked to the loss of T-cells in severe disease remains unclear^11,25^. It may partially reflect the recruitment of lymphocytes to inflamed respiratory vascular endothelium, although lung autopsy studies in certain patients indicate that lymphocytic infiltration is not excessive^26–29^.

In this context, the transcription factor T cell factor 1 (Tcf1, encoded by *Tcf7*) is a critical regulator of T-cell development^30^. TCF1+ progenitor T-cells are a subset of T-cells with the potential to differentiate into various types of T-cells of importance for the immune response against viral infections. TCF1 is expressed in thymic progenitors^31^ as well as naive and various subsets of CD4 and CD8 peripheral T-cells^32^. Further, during chronic viral infections, self-renewing TCF1^+^ progenitors replenish the effector cell pool^33,34^. The genetic knockout of *Tcf7* in CD8^+^ T cells reduces the number and functionality of memory-precursor-like T cells^35^. In particular, these CD8 + T cells sustain the immune response against LCMV^36^ and cytomegalovirus infections^37,38^. Similarly, TCF1+ cells are needed for successful cancer immunology with immune checkpoint inhibitors^35,36,39–42^. Related LEF1 also cooperates with TCF1 to orchestrate CD4+ and CD8+ differentiation^43–45^.

Given the central role of TCF1+ progenitor T-cells in many viral infections, it was surprising that the status of TCF1+ progenitors in SARs CoV2 infections has not been explored in depth. Here, we show that severe COVID-19 disease involves a preferential decline in the presence of TCF1+ progenitor T-cells that alarmingly have lost the capacity to undergo proliferation and self-renewal. Further, sera from severe patients inhibit TCF1 transcription ex vivo which was attenuated by the antibody targeting of the cytokine, interleukin-12 (IL-12). Collectively, our findings suggest that the targeted reduction of TCF1 + T-cells may contribute to the loss of T-cells in severe COVID-19 patients.

## Results

To assess various markers that distinguish severe COVID-19 disease, we studied adults that were hospitalized at the McGill University Health Centre (MUHC) and confirmed by polymerase chain reaction (PCR) to be infected with SARS-CoV-2 [April 2020 and March 2021]. The median time of patient admission to the collection of cross-sectional samples was 3 days (IQR 1–8 days). For longitudinal studies, serial PBMC collections were obtained from 18 individuals at time points out to 36 days from admission. Flow cytometry analysis was conducted on 125 peripheral blood mononuclear cell (PBMC) collections (108 PBMC collections from 76 unique patients and 17 PBMC collections from healthy donors (HD)).

Clinical metadata was available from the COVID-19 patients over the course of their hospitalization (Supplementary Table 1). The cohort studied had a median age of 70.3 years (IQR 59.5–81.7) and was predominantly male (74%). The time covered corresponded to the first and second waves of the COVID-19 pandemic in Montreal, Quebec, Canada which was marked by institutional spread (wave 1), followed by community exposure (wave 2) that preceded the availability of vaccines. The most documented comorbidities in our cohort included hypertension (61%), respiratory disease (38%), cardiovascular disease (34%), diabetes and/or metabolic syndrome (32%), dementia (14%), immunosuppression (13%), active malignancy (11%) and chronic renal disease (9%). Presenting symptoms included fever (55%) and/or respiratory (71%), neurological (42%), gastrointestinal (37%) and cardiovascular (14%) compromise. In-hospital medications included antibiotics (86%), anticoagulants (78%), glucocorticoids (33%), antivirals (12%) and immunomodulators (5%). 28% of patients received advanced organ support (invasive mechanical ventilation (28%), parenteral vasopressors (22%), extracorporeal membrane oxygenation (ECMO) (8%) and/or renal replacement therapy (3%)).

The hospitalized patients were categorized clinically with either mild or severe disease as per their maximal severity scores using the World Health Organization (WHO)’s ordinal scale^46,47^. Of the 51 individuals (67% of the total cohort) who were initially scored as having the mild disease at the time of their initial PBMC collection, 8 (11%) ultimately progressed to severe disease of whom 4 (5%) died from disease complications, whereas 9 of 33 individuals who were initially scored as severe died of disease complications (Supplementary Table 2).

Overall, 66% of our hospitalized cohort were clinically lymphopenic by the time of their first peripheral blood mononuclear cell (PBMC) collection (absolute lymphocyte count < 1000/mL) with 86% having developed lymphopenia at some point during their hospitalization. CD3 + T-cells were gated for the expression of CD4 and CD8 (Supplementary Fig. 1). From this, we noted a progressive decline in the presence of CD3 + T-cells (Supplementary Fig. 2a, upper panel), CD3 + CD4+ (Supplementary Fig. 2b, upper panel) and CD3 + CD8+ peripheral T-cells (Supplementary Fig. 2c, upper panel) in the progression from healthy donor to mild and severe disease. This decline in the presence of cells with increasing disease severity as defined by WOS^46,48^ was confirmed by Spearman analysis (Supplementary Fig. 2a–c, lower panels). These findings are consistent with previous reports of lymphopenia during COVID-19^11,25,47^. Further, the staining of T-cells with an antibody to the TCRβ+ also showed a decrease in cells (Supplementary Fig. 2d). By contrast, the low base line of TCR*γ* + T-cells did not show a loss in cell numbers (Supplementary Fig. 2e). These data confirmed the striking overall loss of peripheral T-cells with increasing COVID-19 disease severity.

Concurrent with this observation was in the expression of activation markers on CD8 and CD4 + T-cells (Supplementary Fig. 2f-i, upper and lower panels, respectively), as reported^5,15,17,49^. This included CD69 in mild and severe disease (Supplementary Fig. 2f), the activation/exhaustion marker, PD1 (Supplementary Fig. 2g), the differentiation antigen, Notch (Supplementary Fig. 2h) and the surface receptor CEACAM1 on CD8 + T-cells (Supplementary Fig. 2i, upper panel). A trend to increase CEACAM1 expression was observed in CD4 + T-cells (Supplementary Fig. 2i, lower panel).

In terms of effector molecules, we observed an increase in the expression of the cytolytic mediator, granzyme B (GzmB) in CD8 cells (Supplementary Fig. 2j, upper panel), which was also correlated with the WOS severity score (Spearman analysis: *r* = 0.3, *p = ) (lower panel). Similarly, cytokine IFNγ1 was increased in CD8 and CD4 T-cells (Supplementary Fig. 2k, l, upper panels, respectively) and correlated with the WOS severity score (lower panels). Examples of viSNE patterns for the different markers (shown in Supplementary Fig. 2m, n). These data confirmed that the increase in COVID-19 severity was accompanied by markers indicative of activation and effector function.

### Preferential loss of TCF1 progenitor T-cells in patients with severe disease

**Fig. 1:**
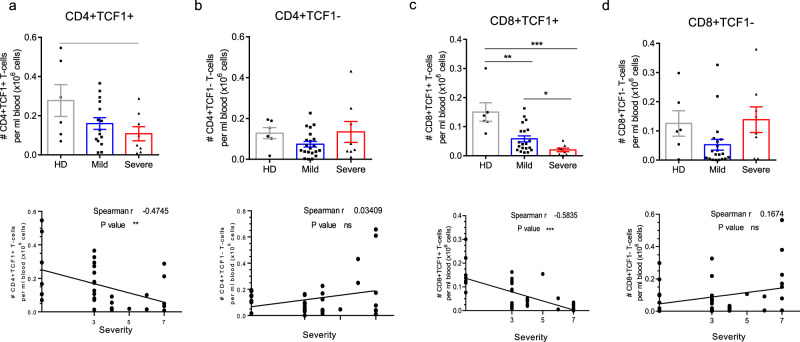
Depicts the reduction of TCF1 + T-cells in severe COVID-19. Peripheral T-cells were extracted from healthy donors (*n* = 12) and patients with mild (*n* = 22) or severe (*n* = 9) disease, followed by an analysis of surface receptors using flow cytometry. **a** The figure demonstrates the preferential loss of CD4 + TCF1 + cells in the blood of severe COVID-19 patients. In the upper panel, a histogram illustrates the decrease in CD4 + TCF1 + cells in a severe patient. The lower panel presents a Spearman analysis showing a correlation between the number of CD4 + TCF1 + cells and disease severity. **b** The figure also showcases the presence of CD4 + TCF1- cells in the blood of COVID-19 patients. The upper panel displays a histogram indicating the continued presence of CD4 + TCF1- cells in a severe patient. The lower panel presents a Spearman analysis showing no correlation between the number of CD4 + TCF1- cells and disease severity. **c** Additionally, the figure highlights the preferential loss of CD8 + TCF1 + cells in the blood of COVID-19 patients. The upper panel shows a histogram demonstrating the reduction of CD8 + TCF1 + cells in a severe patient. The lower panel presents a Spearman analysis showing a correlation between the number of CD8 + TCF1+ cells and disease severity. **d** Furthermore, the figure illustrates the presence of CD8 + TCF1- cells in the blood of COVID-19 patients. The upper panel displays a histogram demonstrating the continued presence of CD8 + TCF1- cells in a severe patient. The lower panel presents a Spearman analysis showing no correlation between the number of CD8 + TCF1- cells and disease severity. The sample sizes for each group were as follows: Healthy Donors (HD) (*n* = 12), Mild cases (*n* = 22), and Severe cases (*n* = 9).

Given this activation of T-cells, it remained a puzzle why there was also a loss of peripheral T-cells, particularly in patients with severe disease. To address this further, it seemed a reasonable hypothesis that progenitor, self-renewing T-cells might be targeted in severe disease^36,37^. In this context, as mentioned, the transcription factor TCF1 defines progenitor stem-like T-cells in the immune system^30^. We therefore initially assessed the numbers of TCF1 + versus TCF1- T-cells per ml of blood of healthy donors (HDs) and patients with mild (M) or severe (S) disease (Fig. 1). From this, we observed a decrease in numbers of CD4 + TCF1 + T-cells per ml with an increasing severity of disease (Fig. 1a, upper panel). Spearman analysis confirmed the decrease in CD4 + TCF1 + cells with increasing disease severity with a WOS ranking of 5–7 (*r* = −0.47; *p*-value < 0.01) (lower panel). This contrasted with CD4 + cells lacking TCF1 (i.e., CD4 + TCF1- T-cells) which showed no obvious change in expression (Fig. 1b, upper panel), an observation also supported by Spearman analysis (lower panel).

Importantly, the same pattern was observed amongst CD8 + T-cells (Fig. 1c, upper panel). In this case, a statistically significant difference was seen between healthy donors and mild diseased samples as well as between mild and severe patients. Spearman analysis confirmed the decrease in CD8 + TCF1 + cells relative to increasing disease severity (*r* = −0.5835; *p* < 0.005) (lower panel). By contrast, no decline in CD8 + TCF1- T-cells was seen (Fig. 1d, upper panel) as confirmed by Spearman analysis (lower panel). These data showed for the first time that TCF1 + CD8 and CD4 + T-cells are preferentially reduced in PBMCs from patients with severe disease.

### Reduced TCF1 and LEF1 expression in T-cells from severe patients

**Fig. 2:**
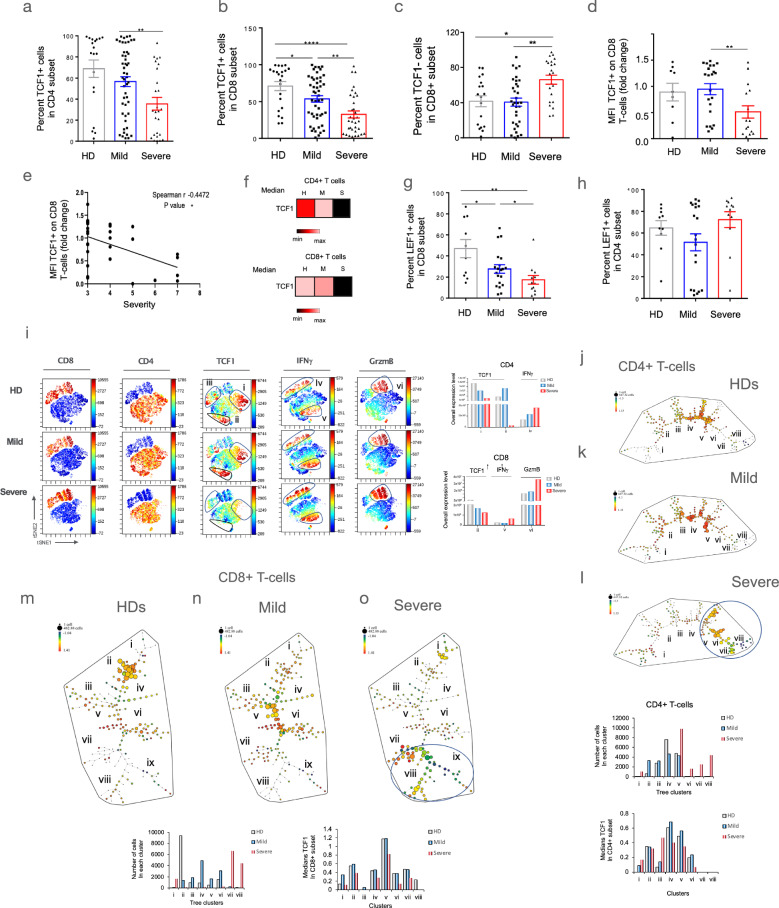
The reduction in the percentage and intensity of TCF1 and LEF1 expression in CD4 and CD8 + T-cells from severe COVID-19. **a** The histogram displays the loss of TCF1+ peripheral T-cells in the CD4 subset in severe patients compared to mild patients. The data is grouped as percentages in mild (WOS 2–4) or severe (WOS 5–8). The sample sizes were as follows: Healthy Donors (HD) (*n* = 19), Mild cases (*n* = 49), and Severe cases (*n* = 26). **b** The histogram shows the loss of TCF1 + peripheral T-cells in the CD8+ subset in severe patients compared to mild patients. The data is grouped as percentages in mild (WOS 2–4) or severe (WOS 5–8). The sample sizes were as follows: Healthy Donors (HD) (*n* = 22), Mild cases (*n* = 49), and Severe cases (*n* = 34). **c** The histogram illustrates the presence of TCF1- peripheral T-cells in the CD8 + subset in severe patients compared to mild patients. The data is grouped as percentages in mild (WOS 2–4) or severe (WOS 5–8). The sample sizes were same as in part b. **d** The histogram presents the Median Fluorescence Intensity (MFI) for TCF1 + expression in CD8 + T-cells from patients with severe disease relative to mild disease. The values are expressed as a fold change of the MFI of peripheral T-cells in the CD8 subset. The sample sizes were as follows: Healthy Donors (HD) (*n* = 9), Mild cases (*n* = 22), and Severe cases (*n* = 16). **e**  rates the reduction of TCF1 + expression as expressed by fold change of the MFI relative to the WOS score. The analysis reveals a negative correlation (*r* = −0.447; *p* =  \*). (*n* = 18). **f** Heat maps depict the reduced MFI for TCF1 in the progression from Healthy Donors (HD) to mild and severe disease. **g** The histogram shows the percentage LEF1 + expression in CD8 + T-cells from Healthy Donors (HD) and patients with severe disease relative to mild disease. The sample sizes were as follows: Healthy Donors (HD) (*n* = 10), Mild cases (*n* = 20), and Severe cases (*n* = 12). **h** The histogram displays the percentage LEF1 + expression in CD4 + T-cells from Healthy Donors (HD) and patients with severe disease relative to mild disease. The sample sizes were same as in part g. **i** viSNE analysis of peripheral T-cells from Healthy Donors (HD) and patients with mild and severe disease. The analysis incorporates concatenated data from 12 patients in each group. Severe disease is characterized by a marked reduction in the presence of TCF1 + in CD4 and CD8 + clusters. In contrast, CD4 and CD8 cells expressing the effector cytokine interferon-gamma and the effector protein granzyme B (GzmB) increase in severe disease. The right panels include histograms showing an overall loss of TCF1 expression in CD4 and CD8 subsets. **j**–**l** SPADE patterns in CD4 + T-cells from Healthy Donors (HD) showing the loss of TCF1 expression in multiple tree groupings found in severe vs mild patients. SPADE analysis identifies over 100 subsets or nodes with varying degrees of similarity seen in each tree branch. The fluorescence intensity of different markers for each node is represented by color, while the size of the node represents the number of cells. The figure displays the concatenated samples from 9 patients in each treatment group involving multiple markers (12 different antibodies). Equal numbers of cells per treatment group were analyzed. The pattern in severe disease shows an increase in the number of nodes with reduced TCF1 expression (dark blue-green color). **m**–**o** SPADE patterns in CD8 + T-cells from Healthy Donors (HD) showing the loss of TCF1 expression in multiple tree groupings found in severe vs mild patients. The figure displays the concatenated samples from 9 patients in each treatment group involving multiple markers (12 different antibodies). Equal numbers of cells per treatment group were analyzed. The pattern in severe disease shows an increase in the number of nodes with reduced TCF1 expression (dark blue-green color). The right histograms display the expression values for each cluster.

Given this, we next examined the expression of TCF1 in more detail by assessing expression in a larger patient cohort of > 90 patients (Fig. 2). This analysis showed a decrease in the mean percentage of TCF1 + T-cells in the CD4 population from patients with severe disease (Fig. 2a). The decline was also seen in CD8 + T-cells with a statistical reduction between HDs and mild patients as well as between severe and mild patients (i.e., from 53 to 32%) (Fig. 2b). By contrast, there was an increase in the relative mean for CD8 + TCF1- T-cells in severe patients (Fig. 2c). We also observed a decrease in the mean fluorescent intensity (MFI) of TCF1 expression in the CD8+ cells from severe vs mild diseased (Fig. 2d). This was underscored by Spearman analysis which showed a decrease in TCF1 expression in CD8+ cells commensurate with disease severity (*r* = −0.47, *p* = *) (Fig. 2e). Heat map analysis further confirmed the reduction in TCF1 expression (Fig. 2f). In addition, SLAMF6, a surrogate for TCF1 expression also declined in CD4 and CD8 T-cells (Supplementary Fig. 3, left panels). Conventional FACs profiles of a representative patient could also be used to show a decline in TCF1 (upper panels; HD = 50.5% vs mild = 48.8% vs severe = 16%) and SLAMF6 expression (lower panels; HD = 35.1% vs mild = 30.3% vs severe = 9.15%) in CD8 + T-cells (Supplementary Fig. 3, right panels).

Further, in keeping with a decline in progenitor T-cells, we also analysed a separate cohort of patients and found a significant reduction in the percentage of cells expressing of another progenitor transcription factor, lymphoid enhancer-binding factor 1 (LEF1) in CD8 + T-cells (i.e., CD8 + LEF1 + ) (Fig. 2g). LEF1 and TCF1 are known to co-orchestrate CD4 + and CD8 + T-cell differentiation^43^. However, unlike TCF1, we did not observe a clear difference in LEF1 expression in the CD4 + population (Fig. 2h). No difference in expression of a related family member ZEB1 in CD4 and CD8 + T-cells (Supplementary Fig. 4). For this reason, we chose to focus on TCF1 expression for the remainder of our study.

Using a bioinformatic approach, we employed high-dimensional viSNE analysis to examine the decline of TCF1 expression in distinct subsets of T-cells from a randomly selected concatenated group of samples comprising 12 patients (Fig. 2i). Concatenation involves combining individual samples from different patients into an averaged value. Our analysis revealed two distinct clusters of CD3 + cells, identified by anti-CD4 and CD8 antibodies (two left panels). Furthermore, anti-TCF1 staining allowed the identification of two subsets of clusters within the CD4 population (clusters i and ii) and one cluster within the CD8 + subset (cluster iii). Notably, mild disease cases exhibited an increase in TCF1 expression in cluster ii. In contrast, severe disease cases were characterized by a significant reduction in all clusters (lower middle panel), as evident from the histograms illustrating an overall loss of TCF1 expression in both CD4 and CD8 subsets. Additionally, we observed a similar pattern in another cohort (Supplementary Fig. 5), where severe patients displayed a pronounced loss of cluster 1 in CD4 (Supplementary Fig. 5a) and CD8 (Supplementary Fig. 5b) subsets, as confirmed by the histograms demonstrating reduced TCF1 expression in clusters 2 and 3.

By contrast, the expression of the effector cytokine, interferon-gamma increased in the CD4 cells (i.e., cluster v) and CD8 cells (i.e., cluster iv). Similarly, we observed an increase in the percentage of CD8 + T-cells expressing granzyme B (GzmB) in samples from patients with severe disease (i.e., cluster vi, also see histogram).

With another bioinformatic approach, spanning-tree progression analysis for density-normalized events (SPADE) was used to analyse CD4 (Fig. 2j–l) and CD8 T-cells (Fig. 2m–o)^50,51^. The gating approach for SPADE analysis and the identification of CD4 and CD8 expressing cells is shown (Supplementary Fig. 6). This approach identified >70 different cell subsets (i.e., nodes). The size of each node depicts cell number while the color denotes the MFI. The CD4 + pattern from healthy donors showed moderate levels and numbers of TCF1 + cells in tree groupings iv and v (Fig. 2j). The pattern in mild patients showed a similar presence in the size and color intensity in tree groupings iv and v (Fig. 2k, also see lower histograms). By contrast, CD4 + T-cells from severe diseased patients showed a marked reduction of TCF1 + cells in tree groupings iv and v accompanied by a new grouping of cells vi, vii and viii (Fig. 2l). Grouping vi showed a moderate reduction in TCF1 expression (i.e., from bright orange to yellow/green). A further decrease was seen in groupings vii and vii. Histograms show a shift in the presence of cells to tree clusters v–viii with lower MFI levels seen in clusters vi–viii (lower panels).

A similar TCF1 decline was observed in CD8 + T-cells (Fig. 2m–o). Notably, samples from healthy donors (HDs) and mild disease cases exhibited comparable levels of TCF1 expression (Fig. 2m vs n). In contrast, severe disease cases demonstrated a notable shift of cells into three distinct groupings (vii, viii, and ix) (Fig. 2o), as illustrated by the lower histograms. Furthermore, tree groupings vii and ix displayed a significant reduction in TCF1 expression, indicated by the blue color (Fig. 2o). The histograms depicted a shift in the distribution of cells towards clusters vii and viii (lower left panel), accompanied by lower mean fluorescence intensity (MFI) levels in clusters vi-viii (lower right panel). Collectively, these findings highlight that severe disease is characterized by a pronounced decrease in the presence of T-cells expressing TCF1.

We conducted an analysis to investigate the presence of TCF1 expression in severe patients with a Vitamin D deficiency (Supplementary Fig. 7). Previous studies have reported a higher occurrence of severe COVID-19 cases in individuals with Vitamin D deficiency^52^. Consistent with these findings, we observed a significant increase in the proportion of severe cases (42%) among patients with Vitamin D levels below 25 nmol/L (panel b). Additionally, we found a statistically significant decrease in the mean percentage of TCF1-expressing cells in samples from Vitamin D-deficient individuals (panel c). This observation provides further evidence of a connection between severe disease and reduced TCF1 expression in a subset of patients with Vitamin D deficiency.

### The loss of TCF1 expression in T-cell subsets

**Fig. 3:**
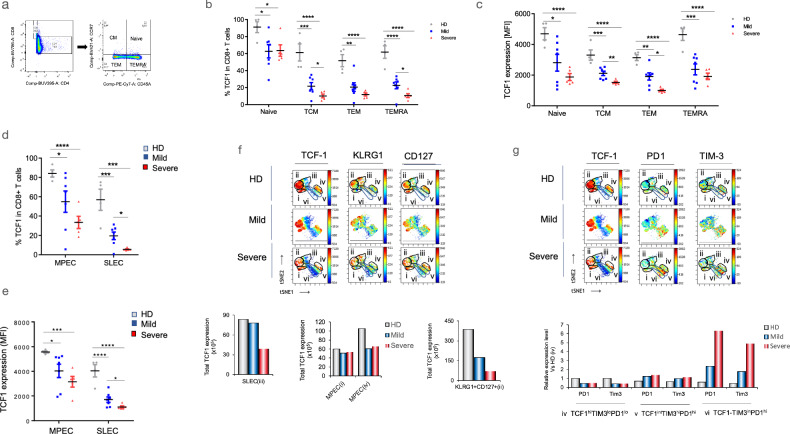
Loss of TCF+ expression in all T-cell subsets with severe COVID-19. Peripheral T-cells were isolated from healthy donors as well as patients with mild or severe disease, and their surface receptors were analyzed using flow cytometry. **a** FACs staining protocol was employed, using CCR7 and CD45RA markers, to identify TCF1 + CD8+ subsets, including naïve (CCR7 + CD45RA+), central memory (TCM, CCR7 + CD45RA-), effector memory (TEM, CCR7-CD45RA-), and effector memory cells re-expressing CD45RA (TEMRA, CCR7-CD45RA+). **b** Histogram demonstrates the reduced presence of TCF1-expressing cells (as a percentage) in naïve, central memory (TCM), effector memory (TEM), and resident effector memory (TEMRA) T-cells derived from healthy donors, mild cases, and severe cases. The sample sizes were as follows: Healthy Donors (HD) (*n* = 4), Mild cases (*n* = 7), and Severe cases (*n* = 5). **c** Histogram displays the reduced expression of TCF1, measured as mean fluorescent intensity (MFI), in naïve, central memory (TCM), effector memory (TEM), and resident effector memory (TEMRA) T-cells from healthy donors, mild cases, and severe cases. The sample sizes were same as in part (**b**). **d** Histogram presents the FACs profiles representing the reduced percentage expression of TCF1 in Short-Lived Effector Cells (SLECs) and Memory Precursor Effector Cells (MPEC) from healthy donors, mild cases, and severe cases. The sample sizes were same as in part (**b**). **e** Similarly, a histogram shows the reduced intensity of TCF1 expression (MFI) in SLECs and MPEC from healthy donors, mild cases, and severe cases. The sample sizes were same as in part (**b**). **f** viSNE profiles of TCF1, KLRG1, and CD127 expression in subsets of T-cells are displayed. Lower histograms depict the total TCF1 expression (i.e., cell number multiplied by expression level) in SLECs (iii) (lower right), MPECs (i and iv), and KLRG1 + CD127+ (ii) cells (lower right panel). **g** viSNE profiles of TCF1, PD1, and TIM3 expression in subsets of T-cells are shown. The lower histogram demonstrates a loss in TCF1 expression (i.e., cell number multiplied by expression level) in exhausted T-cells.

We next assessed TCF1 expression in subsets of peripheral CD8 + cells^53,54^. TCF1 exhibits its highest expression levels in naïve T-cells, but it is also present in other self-renewing T-cell subsets^55,56^. We examined the expression of TCF1 in naïve (TN, CCR7 + CD45RA+), central memory (TCM, CD8 + CCR7 + CD45RA-), effector memory (TEM, CD8 + CCR7-CD45RA-), and tissue-resident effector memory cells re-expressing CD45RA (TEMRA, CD8 + CCR7-CD45RA+) subsets. These subsets were distinguished based on CCR7 and CD45RA staining (Fig. 3a). As anticipated, naïve T-cells exhibited the highest percentage of TCF1 expression (Fig. 3b). This was observed in healthy donors (HD) where 92% of naïve T-cells expressed TCF1. However, in mild and severe disease patients, the percentage of TCF1-expressing naïve T-cells declined. The reduction was even more pronounced in TCM cells, with a decline from 60% positivity in HDs to 23% in mild disease and a remarkable 11% in severe disease (representing a reduction of over 75%). In TEMs, the percentage of TCF1 expression decreased from 56% in HDs to 19% in mild disease and 13% in severe patients. Similarly, in TEMRAs, TCF1 expression decreased from 61% in HDs to 23% in mild disease and 9% in severe disease. We also observed a decline in the mean fluorescent intensity (MFI) of TCF1 expression (Fig. 3c). The naïve subset showed a decrease from an MFI value of 4750 in HDs to 3025 in mild disease and 1990 in severe patients. In the TCM subset, the MFI declined from 3230 in HDs to 2202 in mild disease and 1830 in severe patients. In the TEM subset, the MFI decreased from 3159 in HDs to 1960 in mild disease and 960 in severe patients. The only exception was seen in TEMRA cells, where the decline in MFI was observed in both mild and severe patients. Overall, these findings indicate that increasing disease severity is characterized by a reduction in TCF1 expression and the percentage of cells expressing TCF1 in each T-cell subset.

CD8 + T-cells can also be divided into short-lived effector cells (SLECs) and memory precursor effector cells (MPEC) subsets^57,58^ While SLECs mediate short-term responses, less abundant MPECs develop into long term memory cells. KLRG1 + CD127- define SLECs, while KLRG1^lo^ CD127 + cells correspond to MPECs^53^. KLRG1^+^ effector CD8^+^ T cells lose KLRG1 in becoming memory T cell lineages^53,59^, while CD127 provides long-lasting pro-survival signals^60^. TCF1 expression was reduced in both subsets (Fig. 3d, e), although the decline was most prevalent in the SLEC population where expression was reduced to 6% of cells (Fig. 3d). A reduction of the MFI of TCF1 expression was seen in both subsets (Fig. 3e), but greater in the SLEC population with a statistical difference between mild and severe disease.

To gain further insights, viSNE identified five distinct clusters based on TCF1, KLRG1, and CD127 expression (Fig. 3f). In healthy donors (HDs), higher TCF1 expression was observed in clusters i, ii, and iv. Cluster iii exhibited the KLRG1+CD127lo phenotype, characteristic of SLECs. Notably, the total TCF1 expression, represented by the cell number multiplied against the mean fluorescent intensity (MFI), decreased in severe patients, as indicated by the lower right histogram. Clusters i and iv displayed a KLRG1loCD127 + phenotype, corresponding to MPECs. Within cluster iv, a noticeable reduction in total TCF1 expression was evident (lower histogram). Additionally, a decline in TCF1 expression was observed in the KLRG1 + CD127 + subset, represented by cluster ii (Fig. 3f, lower right histogram). This subset, previously described by others^61^, exhibited the most pronounced decrease in TCF1 expression (lower right histogram). Collectively, these findings further demonstrate the overall reduction of TCF1 expression in SLECs, the MPEC subset, and the KLRG1 + CD127 + subsets of T-cells.

### The loss of TCF1 expression in exhausted-like T-cells

An additional question concerned T-cell exhaustion with many reports of exhausted T-cells in COVID-19 patients^8,10,62^. This event can be identified with several markers that include high levels of the inhibitory receptors PD1 and Tim3^63^. Indeed, we did observe a reduction of TCF1 expression in PD1 + Tim3 + exhausted like T-cells (Fig. 3g). In 12 concatenated samples from severe patients, we defined TCF1^hi^TIM3^lo^PD1^lo^ (cluster iv), TCF1^int^TIM3^hi^PD1^hi^ (cluster v) and TCF1TIM3^int^PD1^hi^ (cluster vi). The numbers of TCF1TIM3^int^PD1^hi^ cells were seen to increase in cluster vi in mild and more in severe patients. There was also a moderate increase in numbers of TCF1^int^TIM3^hi^PD1^hi^ cells and a slight decrease in the numbers of cells in cluster iv TCF1^hi^TIM3^lo^PD1^lo^ in mild and severe patients. These data are compatible with a link between T-cell exhaustion and reduced TCF1 expression. However, reduced TCF1 expression was broader than this since it was also seen in non-exhausted T-cells such as the KLRG1 + CD127 + subset (panel ii) which showed only weak PD1 and TIM3 expression.

### The marked loss of Ki67 expression indicative of a loss of cell division

**Fig. 4:**
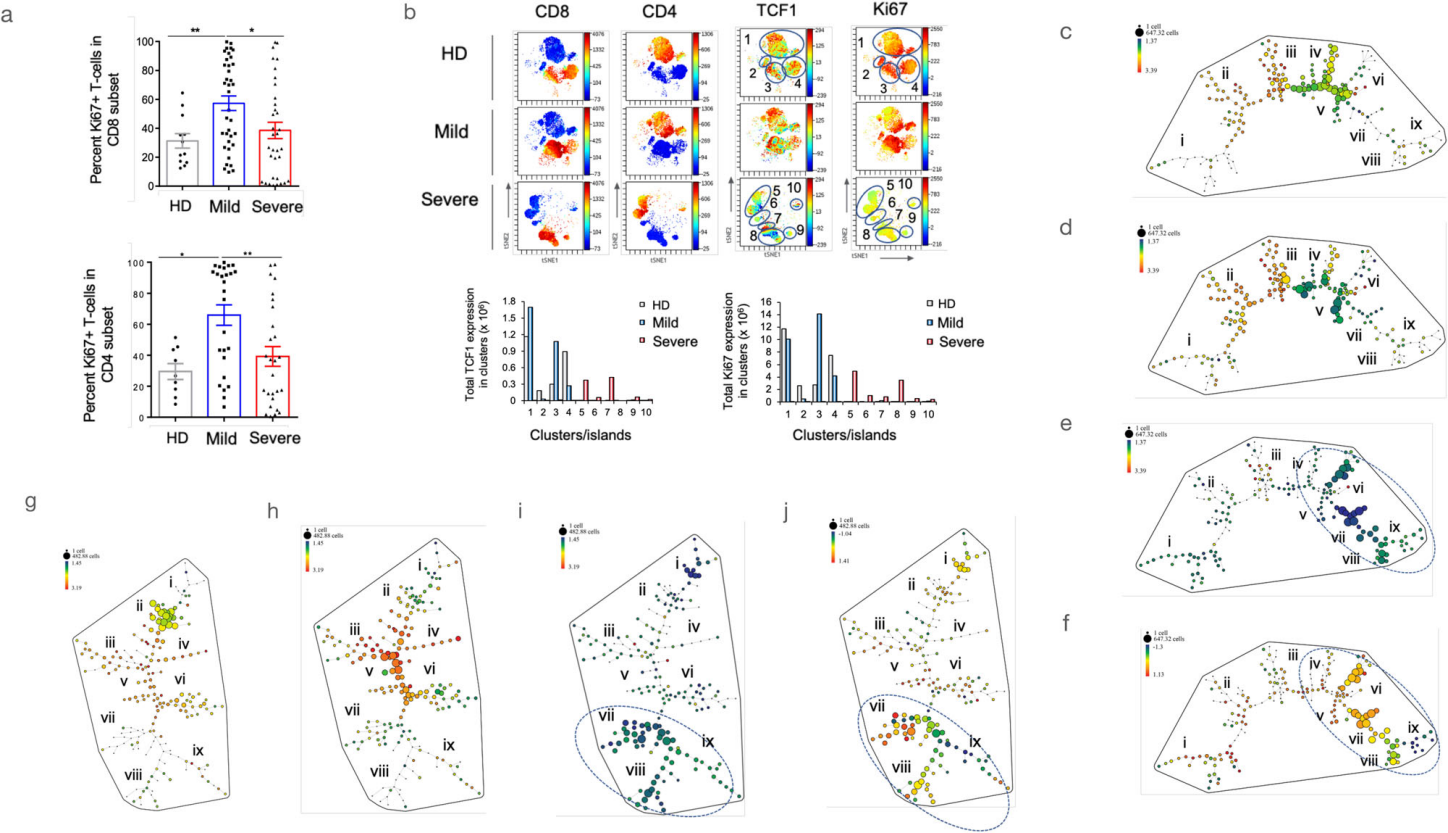
Selective reduction of Ki67 + T-cells in severe COVID-19. Peripheral T-cells were isolated from healthy donors and patients with mild or severe disease, and their surface receptors were analyzed using flow cytometry. **a** The percentage of Ki67 + peripheral T-cells in the CD8 subset was preferentially reduced in patients with mild (WOS 2–4) or severe (WOS 5–8) disease. The sample sizes were as follows: Healthy Donors (HD) (*n* = 12), Mild cases (*n* = 37), and Severe cases (*n* = 34). A similar preferential loss of Ki67 + peripheral T-cells was observed in the CD4 subset. **b** viSNE patterns of anti-Ki67 staining in TCR-beta + T-cells from peripheral blood samples of healthy donors, mild cases, and severe cases are shown. Anti-TCF1 staining is also depicted. Lower panels display total expression histograms for Ki67 (right panel) and TCF1 (left panel). **c**–**e** SPADE patterns illustrate the presence of subsets (nodes) of CD4 + Ki67 + T-cells in healthy donors. SPADE analysis identified over 100 subsets or nodes. Within each pattern, we focused on 9 groupings of tree clusters and their nodes (i-ix). **f** SPADE patterns show the presence of subsets (nodes) of CD4 + TCF1 + T-cells from patients with severe disease. The data is concatenated from 8 patients. **g**–**i** SPADE patterns demonstrate the presence of subsets (nodes) of CD8 + Ki67+ T-cells in patients with severe disease. The data is concatenated from 8 patients. **j** SPADE patterns exhibit the presence of subsets (nodes) of CD8 + TCF1 + T-cells in peripheral cells from patients with severe disease. The data is concatenated from 8 patients. For comparison with (**i**), this pattern displays a heterogeneous collection of nodes with different levels of TCF1 + expression (ranging from light blue to green and yellow), as well as several nodes lacking TCF1 (dark blue). The loss of Ki67 expression overlaps with nodes expressing different levels of TCF1 (indicated by arrows when comparing **j**– **i**).

The loss of TCF1 + T-cells is seen in responses to viral infections; however, this is normally accompanied by progenitor replenishment via cell proliferation. During chronic viral infections in mice, CD8 T cells with high TCF1 exhibit a stem-cell-like phenotype with a better proliferative capacity^36,39,41,64^. It was therefore crucial to assess whether progenitors in COVID-19 could self-renew. For this, we initially monitored cell division using Ki67 expression, a marker for cell cycling^65^. We observed an increase in the mean of Ki67 expression in patients with mild disease from 32–52 percent of CD8 and 32–67 percent of CD4 T-cells (Fig. 4a, upper and lower panels)^5,15,17,49^. However, strikingly, there was an impairment in Ki67 mean expression in CD4 and CD8 + T-cells from severe patients. In fact, the percentage was the same as in patients who had not been infected with SARs CoV2. This indicated that the transition to severe disease was accompanied by a loss in the ability of T-cells [including TCF1 +  progenitors] to self-renew.

Utilizing viSNE analysis on a merged dataset consisting of samples from seven patients, we employed anti-CD4 and CD8 markers to identify distinct cell clusters of CD4 and CD8+ cells (Fig. 4b). Healthy donors (HDs) and mild patients exhibited similar patterns. By contrast, the pattern in severe patient samples had significant changes, characterized by the loss of clusters 1–4 and the emergence of new clusters 5-10. CD3 + CD4+ cells were found in clusters 1, 5, and 10, while CD3 + CD8 + T-cells were present in clusters 3, 4, 7-9. Additionally, a population of CD3 + CD4-CD8- T-cells was observed in islands 2 and 6. Importantly, within clusters 5-10, a reduction in TCF1 expression was observed in all subsets (upper right panels; lower left histogram). CD4 + , CD8 + , and CD4-CD8- cells were all affected. Strikingly, the expression of Ki67, a marker of cellular proliferation increases in mild patient samples compared to healthy donors in clusters 2 and 3. However, in severe patient samples, there was a remarkable loss of Ki67 expression in most clusters (upper right panels; lower right histogram). This loss of Ki67 expression was particularly prominent. Similarly, when analyzing an alternative set of samples comparing mild and severe disease, we observed a significant decrease in cells expressing high levels of Ki67 in cluster 1, accompanied by an increase in the presence of cells with lower levels of expression in cluster 3. This was observed in both the CD4 and CD8 subsets (Supplementary Fig. 8). The mean fluorescent intensity (MFI) values for Ki67 expression further confirmed the loss of expression in clusters 2 and 3 relative to cluster 1 (lower panels).

SPADE analysis further reaffirmed the central observation (Fig. 4c–j). In CD4 cells, a greater number of nodes were observed. Some differences emerged when comparing mild patients to healthy donors, characterized by a partial loss of nodes in tree grouping v, accompanied by an increase in the intensity of expression in tree groupings iv and v (Fig. 4d vs c). In contrast, severe patients exhibited a significant contraction in tree groupings iii-iv, which were replaced by an enlargement of tree groupings v-viii (Fig. 4e). Notably, these enlarged nodes displayed a notable decrease in the expression of Ki67, indicated by dark green and blue colors (circled area). Importantly, this pattern of reduced Ki67 expression coincided with the region displaying reduced TCF1 expression (Fig. 4f vs e). Supplementary Fig. 9 provides examples of CD4 SPADE patterns from individual healthy donors, as well as from patients with mild and severe disease.

A significant reduction in Ki67 expression was also observed in CD8 + T-cells (Fig. 4g–i). Using SPADE analysis, CD8 + T-cells were subdivided into >100 nodes based on 12 concatenated patient samples. These nodes were grouped into tree groupings i–ix. In healthy donors, tree grouping ii exhibited moderate levels of Ki67 expression (Fig. 4g). In mild patients, there was a shift of cells towards groupings iii and vi, which displayed higher levels of expression compared to healthy donors. However, in severe disease, there was a pronounced shift towards groupings vii-ix, accompanied by a substantial decrease in Ki67 expression (Fig. 4i). This pattern aligned with the pattern observed for TCF1+cells with reduced expression (Fig. 4j). Overall, these findings highlight a central point that severe disease is characterized by a significant impairment in the proliferative capacity of T-cells, which is also observed in cells displaying reduced TCF1 expression.

### The loss of TCF1 expression in longitudinal analysis of patients

**Fig. 5:**
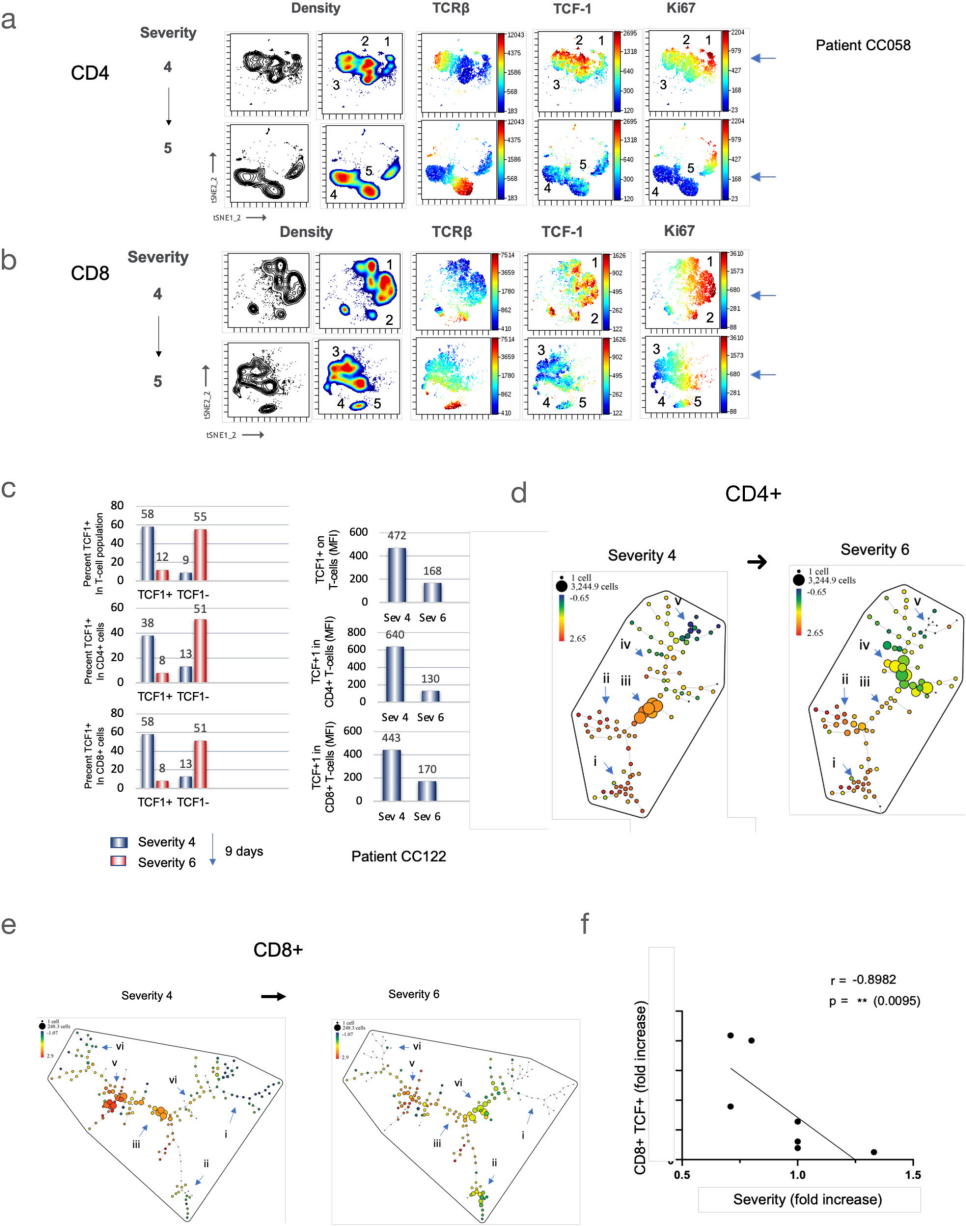
Longitudinal analysis of TCF1 + T-cells with mild to severe COVID-19. Samples were collected from patient CC058, who was infected with SARS-CoV-2 and initially had a severity ranking of 4. After a period of 9 days, a second sample (CC122) was obtained when the patient’s severity score had increased to 6. Peripheral T-cells were extracted from these samples and evaluated for TCF1 expression on both CD4 and CD8 T-cells. **a** ViSNE analysis demonstrates the loss of TCF1 expression in CD4 T-cells as the disease severity progresses from 4–6. **b** ViSNE analysis reveals the loss of TCF1 expression in CD8 T-cells with an increase in disease severity from 4 to 6. **c** The percentage and mean fluorescent intensity (MFI) of TCF1 expression are measured on total T-cells (upper panel), CD4 + T-cells (middle panel), and CD8 + T-cells (lower panel). **d** SPADE patterns show the presence of subsets (nodes) of CD4 + TCF1+ cells longitudinally, comparing a severity ranking of 4 (left grouping) to 6 (right grouping). Based on antibody staining, SPADE analysis identified over 100 subsets or nodes, with relative degrees of similarity observed in each tree branch. The color of each node represents the fluorescence intensity of different markers, while the size of the node indicates the number of cells. Within each pattern, we focused on 5 groupings of tree clusters and their nodes (i–v). The left panel represents severity 4, with TCF1 expression found in tree cluster iii. The right panel represents severity 6, showing reduced TCF1 expression in tree cluster iv. **e** SPADE patterns display the presence of subsets (nodes) of CD8 + TCF1+ cells longitudinally, comparing a severity ranking of 4 (left grouping) to 6 (right grouping). The patterns demonstrate a shift in the numbers of nodes with high to low TCF1 expression. The left panel corresponds to severity 4, with TCF1 expression found in tree clusters iii and v. The right panel corresponds to severity 6, revealing reduced TCF1 expression and cell numbers on nodes in tree cluster iv. **f** Spearman analysis reveals a negative correlation (Spearman’s rank correlation coefficient, *f* = −0.8982) between the decrease in TCF1 + CD8 T-cells (fold change) and disease severity, indicating a significant association (*p* = 0.0095).

We also conducted a longitudinal analysis on a cohort of several patients (*n* = 31) to investigate disease progression (Fig. 5). Taking patient CC058 as a representative example, we obtained an initial sample when the patient was admitted with a severity ranking of 4. Nine days later, a second sample was collected when the patient’s condition had worsened to a severity ranking of 5. viSNE analysis revealed a striking change in the density patterns of cellular expression among CD4 and CD8 + T-cells (Fig. 5a, b). The density pattern of CD4 clusters 1–3 observed during severity 4 transformed into clusters 4 and 5 during severity 5 (Fig. 5a, upper and lower panels). Similarly, CD8 clusters 1 and 2 transitioned into clusters 3–5 in severe disease (Fig. 5b, upper and lower panels).

In CD4 cells, this change was accompanied by an increase in the expression of the TCR-beta chain (Fig. 5a, upper and lower panels). Moreover, the high levels of TCF1 expression observed in clusters 1–3 were markedly reduced in clusters 4 and 5 during severity 5. Similarly, Ki67 expression, which was initially observed in clusters 1–3, was significantly decreased in cluster 4 with a residual presence in cluster 5. Likewise, for CD8 cells, the densitometric pattern underwent significant changes, shifting from clusters 1 and 2 to clusters 3-5 (Fig. 5b). This shift was associated with an increase in TCR-beta expression but a notable decrease in TCF1 and Ki67 expression in clusters 3 and 4. These findings confirmed that increasing disease severity was linked to reduced TCF1 and Ki67 expression in overlapping populations of CD4 and CD8 + T-cells.

In another example, patient CC122 exhibited a significant decrease in the percentage of TCF1 + T-cells from 58% to 12% (Fig. 5c). A similar decrease was observed in the percentage of TCF1 + CD4 and CD8 + T-cells. Conversely, severe patients showed an increase in the representation of TCF1- cells. Additionally, there was a decrease in the mean fluorescent intensity (MFI) values for TCF1 in the overall T-cell population (472 to 168) and in the CD4+ (640 to 130) and CD8+ (443 to 170) compartments (Fig. 5c, upper and lower panels).

SPADE profiles further underscored this change in pattern (Fig. 5d). Over 70 subsets of cells were seen with TCF1 expression in tree grouping iii (left panel). The progression to severity 6 involved the loss of tree grouping iii and the appearance of a new grouping iv with reduced TCF1 expression (right panel). A similar change was seen by SPADE of CD8+ cells expressing TCF1 (Fig. 5e). Nodes with intermediate-high TCF1 expression during the period of severity 4 (iii and v) were lost when the patient progressed to severity 6 accompanied by an increase in nodes with low expression with tree grouping vi. Overall, these representations showed that a diversity of intermediate-high TCF1 expressing CD4 and CD8 cells was greatly reduced with increasing disease severity. Importantly, Spearman analysis conducted on a patient cohort revealed a striking fold decrease in CD8 + TCF1 + T-cells, demonstrating a strong negative correlation with disease severity (*r*-value = −0.8982, *p*-value = 0.0095) (Fig. 5f).

### Bcl-2 and the appearance of caspase 3

**Fig. 6:**
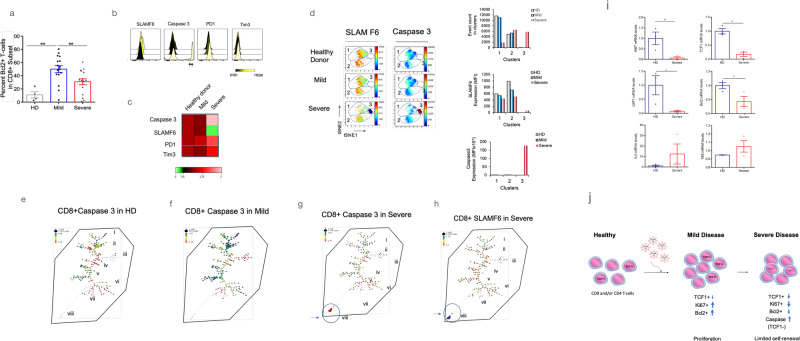
Reduced TCF1+ expression correlated with the induction of caspase 3 expression in TCF1- CD8 + T-cells. **a** Peripheral CD8 + T-cells with reduced Bcl2 expression were observed in patients with severe disease. The samples were categorized into mild cases (WOS 2–4) or severe cases (WOS 5-8), with sample sizes of Healthy Donors (HD) (*n* = 4), Mild cases (*n* = 16), and Severe cases (*n* = 16). **b** The standard FACs profile displayed the expression of markers SLAM F6 (surrogate for TCF1), caspase 3, PD1, and Tim3. SLAMF6 expression was reduced in severe CD8+ samples, accompanied by the appearance of a new peak indicating high caspase 3 expression. PD1 and Tim3 showed a mild increase in expression in severe samples. **c** The heat map, constructed using 12 concatenated samples, demonstrated the marked loss of SLAMF6 expression concurrent with an increase in caspase 3 expression. PD1 and Tim3 showed a milder increase in expression in severe samples. **d** ViSNE analysis of a representative sample from healthy donors and patients with mild or severe disease revealed a decrease in SLAM F6 expression in severe patients (clusters 1 and 2), along with the emergence of a SLAM F6-negative cluster (cluster 3). Cluster 3, characterized by the absence of TCF1 expression, exhibited high levels of caspase 3 in severe patients. The right panel histogram depicted the expression of caspase 3 in different clusters. **e** SPADE patterns illustrated the presence of subsets (nodes) of CD8+ caspase 3 + T-cells from healthy donors (concatenated from 8 patients). **f** SPADE patterns displayed the presence of subsets (nodes) of CD8+ caspase 3 + T-cells from patients with mild disease (concatenated from 8 patients). **g** SPADE patterns demonstrated the presence of subsets (nodes) of CD8+ caspase 3 + T-cells from patients with severe disease (concatenated from 8 patients). The circled cells indicated the appearance of a population of cells expressing high levels of caspase 3. **h** SPADE patterns showed the presence of subsets (nodes) of CD8 + SLAMF6 + T-cells from patients with severe disease (concatenated from 8 patients). The circled cells indicated the appearance of a population that had lost TCF1 expression. **i** Real-time quantitative PCR analysis of the transcription profiles of T-cells from peripheral blood compared healthy donors to severe patients. Severe patients exhibited a significant decrease in the expression of *Ki67*, *TCF1*, *LEF1*, and *Bcl2*, while *IL-6* showed an increased expression. 18 S mRNA served as a control, showing no change in expression. The sample sizes of Healthy Donors (HD) (*n* = 3), Severe cases (*n* = 3). **j** The model demonstrated the increase in TCF1, Ki67, and Bcl2 expression in mild disease, followed by a marked reduction in severe disease, accompanied by the emergence of caspase 3 high-expressing cells lacking TCF1 expression. Ki67 expression increased in T-cells from patients with mild disease but experienced a significant decline in T-cells from severe patients, indicating a loss of self-renewal.

We next asked whether the loss of TCF1+ and Ki67 was associated with an increase in cell death. In this context, B-cell lymphoma-2 (Bcl2) is a pro-survival factor needed for the survival of stem cells and T lymphocytes^61^. With a focus on the CD8 + T-cells, we found that an increase in Bcl2 expression in patients with mild disease relative to HDs (Fig. 6a). By contrast, we observed a significant reduction of Bcl2 expression in CD8 cells in patients with severe disease compared with mild disease. A reduction was also seen by standard FACs profiling of the expression of SLAMF6, a surrogate marker for TCF1 expression (Fig. 6b). An example is shown with a reduction in expression in SLAMF6 accompanied with an increase in PD1 and TIM3 expression. A similar reduction was seen in a heat map in analyzing a cohort of 12 patients (Fig. 6c). This showed a decrease in SLAMF6 expression accompanied by a slight increase in PD1 and TIM3 expression, together with a marked increase in the expression of the cysteine-aspartic acid protease, caspase 3, a marker for cell death^66^ (Fig. 6b, c). In this context, cell death would be expected to contribute to the decline of T-cells seen in patients with severe disease^67^.

We next assessed whether this event affected a particular subset of cells (Fig. 6d). In terms of viSNE profiling, the loss of cells in SLAMF6+ clusters 1 and 2 in the severe disease pattern was accompanied by the appearance of a new cluster 3, which showed no SLAMF6 staining (Fig. 6d). Instead, this SLAMF6 negative cluster stained brightly for caspase 3, an indicator of cell death (lower right panel). Histogram profiles showed a shift in event counts (i.e., numbers of cells) from clusters 1 and 2 to cluster 3 (upper right histogram).

SPADE analysis of CD8 + T-cells provided additional information by showing the expression of low levels of caspase 3 expression nodes in CD8+ cells from healthy donors (Fig. 6e) and mild disease (see tree groupings i, ii, iv and v) (Fig. 6f). The larger negative nodes in mild patients are consistent with an increase in the number of cells. By contrast, severe patients showed a noticeable contraction of numbers of cells accompanied by the appearance of a large node at the bottom of the pattern (Fig. 6g see arrow). The same node failed to express TCF1 and showed a high level of caspase-3 expression (Fig. 6h, see arrow). These data are consistent with the loss of TCF1 expression accompanied by the cell death in a portion of peripheral CD8 + T-cells in patients with severe disease.

Lastly, T-cells from HDs and severe patients were assessed for TCF1 expression by real time PCR (Fig. 6i). Two-step real-time PCR was used to separate the reverse transcription reaction from the real-time PCR assay^68^. In this case, we observed a major decline (i.e., 7-fold) in the transcription in *Ki67*, *TCF1*, *LEF1* and *Bcl2* in T-cells from severe patients relative to HDs. By contrast, as a positive controls, the proinflammatory cytokine *IL6* showed an increase in transcription and 18 S showed no change in the expression in the samples. These data confirmed that Ki67, TCF1 and LEF1 are markedly decreased in expression at the transcriptional level in severe disease. This led to a model where severe disease can be distinguished from mild disease by the expression of markers TCF1, Ki67, Bcl2 and caspase 3 (Fig. 6j).

### Proinflammatory cytokine IL-12 in sera contributes to TCF1 downregulation

Lastly, COVID-19 severe disease is associated with a severe cytokine storm^69,70^. Although findings vary from study to study, the increase in severity has been reported to involve pro-inflammatory interleukins such as IL1α, IL1β, IL6, IL8, IL12, IL17, and TNFα (tumor necrosis factor-α)^22,69,70^. IL12 was of special interest to us given than it downregulates TCF1 at the transcriptional level^71^. To assess whether IL12 in the plasma of severe patients was responsible for the loss of TCF1+ expression in T-cells, we first measure the levels of IL12 in the sera of severe patients relative to healthy donors and as expected found increased levels of the cytokine over a range of 100–400 pg/ml (Supplementary Fig. 10a), consistent with previous reports^8,9,20,21,69^. We then incubated peripheral T-cells from healthy donors with aliquots of sera (10%) from healthy donors or severe patients for 6 days followed by an assessment of TCF1 expression (Supplementary Fig. 10b). From this, we found the sera from severe patients reduced TCF1 expression in CD8 and CD4 + T-cells (Supplementary Fig. 10b, upper and lower panels). Importantly, the co-incubation of a blocking antibody to IL12p40 (5μg/ml) reversed this decrease in expression (Supplementary Fig. 10b). ViSNE analysis of CD8 + T-cells confirmed the reduction in TCF1 expression as seen in clusters 1,2 and 3 (left panels) while the anti-IL12 antibody reversed this effect (Supplementary Fig. 10c, also see right histograms). Finally, the loss of TCF1 expression was observed in the naïve, TCM, TEM and TEMRA cells and was reversed by anti-IL12 in all subsets (Supplementary Fig. 10d). This data indicates that IL12 in the severe sera can contribute to the reduced expression of TCF1 in T-cells from patients with severe disease.

## Discussion

The mechanism responsible for T-cell lymphopenia in severe COVID-19 have yet to be fully understood. Our study sheds light on this issue with several key findings. Firstly, it demonstrates a significant and preferential decline in TCF1+ progenitor T-cells crucial for the effective response of peripheral T-cells against infections. Further, the remaining TCF1 + T-cells also showed impaired self-renewal with a loss of Ki67 expression. This decline was observed in both CD4 and CD8 + T-cells, encompassed various subsets such as naïve and tissue-resident effector memory cells, as well as SLECs and MPECs. Further, severe COVID-19 patients exhibited reduced expression of the pro-survival factor BcL2 and the emergence of a TCF1-low, caspase 3 + T-cell population, indicating increased cell death.

Additionally, our study revealed that sera from severe COVID-19 patients inhibited TCF1 transcription ex vivo, an inhibition that could be counteracted by blocking antibodies against IL12. This suggests a role of IL-12 in the decline of TCF1+ progenitor T-cells in severe COVID-19 cases. Overall, this study provides valuable insights into the mechanisms underlying the development of severe COVID-19 and emphasizes the significance of TCF1+ progenitor T-cells in the immune response against the virus. In our model, we predict that the cytokine storm would precede the loss of T-cells in the peripheral immune system where IL-12 (and possibly other cytokines) inhibit TCF1+ transcription leading to the preferential loss of dividing progenitor T-cells that are needed to replenish the immune system. It also suggests a treatment option for severe disease involving blocking antibodies against IL12 or related cytokines that down-regulate TCF1.

As reported by others^9,10,12,17,23,24^, we first noted the major loss of peripheral CD4 and CD8 T-cells with increasing disease severity. In the same instance, when comparing the presence of TCF1+ versus TCF1- cells, we observed an unexpected preferential reduction in CD8 + TCF1 + T-cells from the peripheral blood of severe patients which was statistically correlated with increasing WOS severity scores. A reduction in the MFI and the percent representation of TCF1 + CD8 T-cells in peripheral blood was seen. We noted this reduced expression in both cross-sectional and longitudinal analysis and in different CD8+ subsets. As reported^8^, we also observed an increased expression of markers indicative of T-cell exhaustion (i.e., PD1/TIM3hi). TCF1 expression was not apparent in transitory or terminally exhausted CD8+ T-cells^50^. Further, we observed a reduced TCF1 expression in the naïve, TCM, TEM, TEMRA, SLEC and MPEC subsets. TCF1 favors the development of memory CD8 T cells in Listeria infection^72^. CD8+ high TCF1 T-cells also exhibit a stem-cell-like phenotype with a self-proliferative capacity^36,39,41,64^.

We also noted a surprising concurrent reduction in the expression of Ki67 in both TCF1+ and TCF1- CD8+ T-cells in severe disease. This was a surprising finding and apparent in over 75% of patients with severe disease. Ki67 is a well-established marker for cell cycling^65^ and used as a measure of proliferation in tumors, determining treatment decisions^73^. We found that T-cells from mild disease patients actually showed an increase in expression indicative of a productive response against SARs CoV2. This is consistent with a previous study showing Ki67 expression on subsets such as the CD8+ subset CD38 + HLA-DR+Ki67+^12^.

However, in patients with increasingly severe disease, Ki67 expression was drastically reduced in CD4 cells, and especially in the CD8 subset, as depicted in viSNE and SPADE analysis. In viSNE, there was a clear displacement of cells from a cluster with high Ki67 expression to a new cluster displaying considerably lower expression. CD8 + T-cells experienced a loss of expression in 98 out of 105 nodes as seen in the SPADE pattern. Further, much of the loss of Ki67 coincided with the nodes showing diminished SLAMF6 or TCF1 expression. Whether the loss of TCF1 itself caused the reduction in Ki67 expression, or vice versa, or are independently regulated, remains to be clarified. In the end, the decrease in TCF1 + T-cells and their self-renewal would be expected to contribute to the decline in the number of T-cells needed to effectively combat the virus. TCF1 and associated memory are pivotal in maintaining protective immunity^36,38^.

In addition, we observed a decrease in the expression of the pro-survival mediator BcL2, which belongs to a family of mediators involved in regulating cell survival^74^. Although this reduction was less pronounced compared to TCF1 and Ki67, it was still statistically significant in the severe disease group. In this context, qPCR analysis further confirmed a significant loss in mRNA expression of *TCF1*, *BcL2*, and *Ki67*. Similar to Ki67, BcL2 expression actually increased in mild patients indicating an augmented cell survival in response to infection. However, in severe patients, some altered event occurred that led to a decline in BcL2 expression in CD8 + T-cells. Additionally, in many patients, we observed the emergence of CD8+ cells lacking TCF1 expression, which was accompanied by an increase in caspase 3 expression, an indicator of cell death. While the overall loss of TCF1/Ki67 expression affected the majority of T-cells, further studies will be needed to identify the effect on specific SARs COV2 reactive T-cells and T-cells reactive against other pathogens. Unfortunately, there are now few patients in a hospital setting with severe COVID-19 disease.

It is noteworthy that there were also differences in the response of CD4 and CD8 + T-cells. We observed a significant loss of LEF1 in CD8 + T-cells, another progenitor transcription factor^44^. which was unaltered in CD4 + T-cells from severe patients. LEF1 and TCF1 orchestrate CD4 + T_FH_ differentiation and the loss of either will impair CD4+ cell differentiation^43^ TCF1 and LEF1 also specify T cell lineage and β-selection in thymic differentiation^31^. However, unlike LEF1, TCF1 promotes the differentiation of the CD4 + T_H_2 subset helper T cells^75^. Similarly, Notch 1 signaling is required for the commitment of thymic T-cell progenitors^76^. LEF1 and Notch 1 were able to distinguish severe from mild COVID-19 in the CD8 subset.

In addition, we conducted an analysis to investigate the presence of TCF1 expression in severe COVID-19 patients with vitamin D deficiency. Individuals with a vitamin D deficiency have been reported to experience more severe cases of COVID-19^52^. Consistently, we observed a higher prevalence of vitamin D deficiency in hospitalized COVID-19 patients (50 out of 366, 14.9%) compared to a control group of non-hospitalized individuals without COVID-19 (16 out of 1000, 1.6%) (odds ratio OR 10.7, 95% confidence interval CI 6.0-19.1). Moreover, we found a significant increase in the frequency of severe disease (~42%) among patients with vitamin D levels below 25nmol/L. Within this group, we noted a decrease in the mean percentage of TCF1-expressing cells. Previous studies have reported an interaction between the vitamin D receptor with TCF1 or LEF1^45,77^.

Finally, we discovered that IL-12 in sera from severe patients inhibited the transcription of TCF1 in normal T-cells. Importantly, this effect was prevented with anti-IL12 blocking antibodies. This finding aligns with the showing that IL-12 downregulates TCF1 transcription, partly by inhibiting DNA methyltransferases^71^. Further, lymphopenia in severe disease has been correlated with higher levels of inflammatory cytokines such as IL-12^8,9,20,21^. In addition, the expression of TCF1 was reduced in various subsets of CD8 cells, including naïve, central memory, effector memory, and TEMRs were affected by IL-12. IL-12 is generated by antigen-presenting cells such as dendritic cells (DC) as well as monocytes, macrophages, neutrophils, natural killer (NK) cells and subsets of T-cells. It is therefore likely produced by the activated DCs and macrophages during SARs CoV2 infection. We would now argue that the sustained presence of IL-12 (and possibly other cytokines) during infection leads to the sustained downregulation of TCF1 and the loss of progenitor T-cells needed to sustain the pool of effector T-cells needed to mount a response against the virus and other infections.

Overall, our study suggests that the use of anti-IL-12 blocking antibodies such as Stelara (ustekinumab), presently used to treat psoriasis and Crohn’s disease (CD)^78^ might also help to prevent the loss of T-cells in severe disease. Further, in this context, the anti-inflammatory glucocorticoid, dexamethasone, which has proven successful in treating COVID-19 patients^79^, also inhibits IL12 production^80^. While the use of IL12 in combination with Spike protein vaccines might help promote initial T-cell responses (NCT04627675), it might be of concern later in disease progression. Whether other inflammatory cytokines also contribute to the loss of TCF1 + T-cells and disease severity awaits further studies.

## Materials and methods

### Patients and clinical data collection

This study was conducted in accordance with the ethical guidelines set by the McGill University Health Centre Research Institute (MUHC-RI) Ethics Board, under the approved study protocol (#2021-6081). Informed consent was obtained from patients admitted to the McGill University Health Centre (MUHC) with confirmed SARS-CoV-2 infection between April 2020 and March 2021 (*n* = 76). Peripheral blood samples, including K2EDTA-preserved whole blood and/or sera, were collected from both the patient cohort and healthy adult donors (HDs) who had no prior diagnosis of COVID-19 or recent symptoms consistent with the disease. Detailed clinical data were recorded using standardized case report forms. The median time between patient admission and the collection of initial peripheral blood mononuclear cell (PBMC) samples was 3 days (interquartile range ${{{{\backslash }}}}[{{{{{\rm{IQR\backslash }}}}}}]$ :1–8 days). Serial samples from a subset of patients (*n* = 18) were collected at multiple time points, up to 36 days from admission. The severity of COVID-19 manifestations and clinical outcomes were assessed using the World Health Organisation (WHO)‘s COVID ordinal scale ${{{{\backslash }}}}[{{{{{\rm{r}}}}}}{{{{{\rm{eference\backslash }}}}}}]$. Scores of \<=4 were categorized as mild manifestations, while scores ≥5 were considered severe. Clinical laboratory data were collected from time points that were closest to the research blood collection, as well as time points associated with extreme values. Additional peripheral blood samples from uninfected healthy adults were obtained from the Hema-Quebec blood bank, following ethical approval by the CR-HMR Ethical Approval (Le Comité de protection des animaux du CIUSSS de l’Est-de-l'Île-de-Montréal ${{{{\backslash }}}}[{{{{{\rm{CPA}}}}}}-{{{{{\rm{CEMTL\backslash }}}}}}]$, F06 CPA-21061 du projet 2017-1346, 2017-JA-001). All ethical regulations relevant to human research participants were followed.

### Sample processing

To isolate peripheral blood mononuclear cells (PBMCs), the density gradient centrifugation method was employed at the MUHC-RI. Peripheral blood was collected into K2 EDTA tubes (BD) and carefully layered above an appropriate volume of density gradient medium in 15 ml tubes (e.g., Lymphoprep by Stemcell Technologies). The tubes were centrifuged at room temperature (RT) for 30 min at 400 g. Subsequently, the plasma fraction was collected and stored. The PBMC layer, located between the plasma and the density gradient medium, was carefully collected and washed twice with phosphate-buffered saline (PBS). The isolated PBMCs were then stained for viability, counted, and fixed in 2% paraformaldehyde at RT for 20 min to inactivate the SARS coronavirus^70^. After fixation, the PBMCs were washed twice with fluorescence-activated cell sorting (FACS) buffer (PBS containing 2% fetal bovine serum), centrifuged (400 g, 5 min, RT), and finally frozen in freezing media (90% FBS/10% DMSO) at −80 °C until further FACS staining.

### Antibody panels and staining

On the day of FACs staining, the thawed PBMCs were washed with PBS containing 2% FBS (FACs buffer). The PBMCs were then stained with the following antibodies: AF700-conjugated anti-CD3 (clone: UCHT1), APC-conjugated anti-TCR alpha/beta (clone: IP26), FITC-conjugated anti-TCR gamma/delta (clone: B1), BV785-conjugated anti-CD8 (clone: RPA-T8), BUV395-conjugated anti-CD4 (clone: RPA-T4), BV605-conjugated anti-PD-1 (clone: EH12.1), BV650-conjugated anti-Notch (clone: MHN1-519), and APC-Cy7-conjugated anti-CD69 (clone: FN50). The staining was performed in FACs buffer for 20 min at 4 °C in the dark. Subsequently, the cells were washed and fixed in Fixation/Permeabilization Buffer for 45 min at 4 °C in the dark. After fixation, the cells were further stained with the following antibodies: PE-conjugated anti-TCF1 (clone: 7F11A10), PEcy7-conjugated anti-IFNg (clone: 4 S.B3), PE-CF594-conjugated anti-Granzyme B (clone: GB11), FITC-conjugated anti-Ki67 (clone: 11F6), and BV421-conjugated anti-Bcl2 (clone: 100). This staining was performed for 30 min in Permeabilization Buffer. In cases where caspase 3 staining was performed, the PBMCs were washed with FACS buffer, spun at 1700 rpm for 5 min, and then stained with the FITC active Caspase-3 apoptosis kit. After staining, the cells were washed again with FACS buffer (1700 rpm, 5 min, RT). Compensation was performed using UltraComp eBeads (ThermoFisher, catalog no. 01-2222-42). The acquisition of stained cells was carried out using a BD LSRFortessa X-20 flow cytometer and DIVA software (Beckton Dickinson). FACS analyses were performed using FlowJo software or Cytobank, including viSNE analyses. It is worth noting that the anti-TCR gamma/delta antibodies were generously provided by Dr. Naglaa Shoukry (CR-CHUM, Montreal).

### Flow cytometry and high-dimensional data analysis

Samples were acquired on a five-laser BD FACS Symphony A5. Standardized SPHERO rainbow beads (Spherotech, catalog no. RFP-30-5A) were used to track and adjust photomultiplier tubes over time. UltraComp eBeads (ThermoFisher, catalog no. 01-2222-42) were used for compensation. Up to 2 × 10^6^ live PBMCs were acquired per sample. viSNE analyses were performed on Cytobank (https://cytobank.org). In this case, CD4 T cells, and CD8 T cells were analyzed separately. viSNE analysis was performed using equal sampling of 1000 cells from each FCS file, with 3000 iterations, a perplexity of 60, and theta of 0.5. The following markers were used to generate the viSNE maps: CD8, CD4, CD44, TCF1, PD1, CD69, CD45RA, CCR7, KLRG1, CD127, Ki67, IFNg, GzmB, SLAMF6, Caspase3.

### Longitudinal analysis and correlation plots

To assess samples from participants over time, we sampled the same patients a time after admission to the hospital and during the monitoring of the severity of the disease. Pairwise correlations between variables were calculated and visualized as a correlogram using the R function complot. Spearman’s rank correlation coefficient (r) was indicated by square size and heat scale; significance was indicated by *P < 0.05, **P < 0.01, and ***P < 0.001; and a black box indicates a false- discovery rate (FDR) < 0.05.

### Quantitative real-time PCR

Total RNA was isolated from the PBMCs and reverse-transcribed to obtain cDNA using All-In-One 5X RT MasterMix (Applied Biological Materials Inc, BC, Canada). Real-time PCR amplification of the cDNA was analyzed using BrightGreen 2X qPCR MasterMix-Low ROX (Applied Biological Materials Inc, BC, Canada) and QS12K Flex (Bio-Rad Laboratories, Hercules, CA, USA) ABI QS12K Flex system (Thermo Fisher Scientific). The results were analyzed using the CFX Manager software and normalized to the levels of the housekeeping gene. The primers for qPCR analysis are shown, human-*18S*-F (GATTAAGTCCCTGCCCTTTGT), human-*18S*-R (GTCAAGTTCGACCGTCTTCTC), human-*TCF7*-F(CTGACCTCTCTGGCTTCTACTC), human-*TCF7*-R(CAGAACCTAGCATCAAGGATGGG), human-*Ki67*-F(TGCTCTGGGTTACCTGGTCT), human-*Ki67*-R(GGCTTCTCCCCTTTTGAGAG), human-*LEF1*-F(CTTGTCTGGTAAGTGGCTTCTC), human-*LEF1*-R(ACAGAGTGGGTTTGGCTATTAC), human-*IL6*-F(AGACAGCCACTCACCTCTTCA), human-*IL6*-R(CACCAGGCAAGTCTCCTCATT), human-*Bcl2*-F(TGGATGACCGAGTACCTGAACCG), and human-*Bcl2*-R(TGCCTTCAGAGACAGCCAGGAG).

### Isolation and culture PBMCs with human sera and anti-IL12

Fresh PBMCs were isolated from blood obtained from Hema-Quebec in Quebec. The isolated PBMCs were then allowed to rest overnight in RPMI 1640 medium (Corning, USA) supplemented with 10% heat-inactivated fetal bovine serum (Gibco, USA), penicillin (100U/ml, Hyclone, USA), and 2-Mercaptoethanol (50 μM, Sigma, USA) at 37 °C. On the following day, the PBMCs were stimulated with 10% healthy or severe sera. This stimulation was performed in the presence or absence of a neutralizing monoclonal antibody to IL12p40 (5 μg/ml, R&D) or an isotype control antibody (Monoclonal Mouse IgG1 Clone # 24901). After 6 days of culture, the cells were resuspended in staining buffer, which consisted of PBS with 2% FBS. Surface antibodies (anti-CD3, CD4, CD8) and an intracellular antibody (anti-TCF1) were added to the cell suspension and incubated for 30 min in the dark at 4 °C. Subsequently, the cells were washed twice with staining buffer and resuspended for analysis using a flow cytometer.

### Vitamin D analysis

25D levels were measured on prospectively collected blood samples from 336 patients (March 2020 – January 2022) requiring hospitalization for COVID-19. For the purposes of comparison of vitamin D status between the general population and individuals with COVID requiring hospitalization, the COVID cohort was matched by age & sex against a cohort of 1000 outpatients with bloodwork done as part of routine clinical management over a 2-year period (2018–2019). Definition of vitamin D status: vitamin D sufficiency: [25D] >75 nmol/L; vitamin D insufficiency: [25D] 25-75 nmol/L; vitamin D deficiency: [25D] <25 nmol/L. Differences among groups were assessed using chi-square test for categorical variables and *t*-test or ANOVA for continuous variables.

### Statistics and reproducibility

Individual analyses and sample sizes are described in figure legend sections. All data are expressed as mean ± SEM. A *t*-test was used when only two groups were compared, and a one-way ANOVA was used when more than two groups were compared. A two-way ANOVA was used for multiple comparison procedures that involved two independent variables. A difference in mean values between groups was significant when *p ≤ 0.05; **p ≤ 0.01 and ***p ≤ 0.001.

AIChatGPT was used to compare some text compared to our original text. In some cases, if the literacy of the description was improved, the AI version was partially used while in other cases it was rejected. No content was altered in the paper.

### Reporting summary

Further information on research design is available in the Nature Portfolio Reporting Summary linked to this article.

### Supplementary information

Supplementary Information

Description of Additional Supplementary Files

Supplementary Data

Reporting Summary

## Data Availability

All data, code, and materials used in the analyses will be available to any researcher for purposes of reproducing or extending the analyses. The underlying source data for this study can be found in the Supplementary Data file.
