# Supplementary material for: Decreased progenitor TCF1 + T-cells correlate with COVID-19 disease severity
Source: Commun Biol. 2024 May 3;7:526. doi: 10.1038/s42003-024-05922-2 (PMC11068881; doi:10.1038/s42003-024-05922-2)
Supplement: Supplementary file 1 — Supplementary Information [file 42003_2024_5922_MOESM1_ESM.docx]

­­­­­ Supplementary information for:

**Decreased Progenitor TCF1+ T-Cells Correlate with COVID-19**

**Disease Severity**

Thai Hien Tu^1-3^, Ami Grunbaum^4-6^, François Santinon^1-3^, Alexandra Kazanova^1-3^, Nicholas Rozza^4,5^, Richard Kremer^4-6^, Catalin Milhalcioiu^7^ and Christopher E. Rudd^1-4*^

^PDF file includes:^

^Captions for Supplementary Tables S1-S2^

^Supplementary Figures: S1-S10^

Supplementary Table 1.

Supplementary Table 2.

Supplementary Figure 1

**Supplementary figure 1:** Gating strategy for flow cytometry analysis of blood lymphocytes. PBMCs were isolated as described and stained with various antibodies and analyzed by FACS. An initial acquisition gate on the FSC versus the SSC profile that excluded most of the non-lymphocytes was introduced to minimize background fluorescence. Single cells were gated based on their forward scatter height and area, and alive cells within the single cell gate were identified as BV510 negative population. T cells were identified using an anti-CD3 Ab. CD4 + cells and CD8 +cells were then gated and screened for the expression of other markers indicated in study. FCS = forward scatter, SSC = sideward scatter, -A = area, -H = height.

Supplementary Figure 2

**Supplementary figure 2:** **The loss of T-cells in patients with mild and severe COVID-19.**

a) Peripheral T-cells were isolated from healthy donors, as well as patients with mild or severe disease, and subsequently subjected to flow cytometry analysis of surface receptors. The upper panel of Figure (a) displays a histogram illustrating the percentage of T-cells within the peripheral blood mononuclear population, showing a loss of T-cells in both mild and severe patients. The lower panel demonstrates a Spearman analysis indicating the correlation between the loss of T-cells and the relative severity score, based on a sample size of 8.

b) Similar to panel (a), Figure (b) focuses on the percentage of CD4+ T-cells within the peripheral blood mononuclear population. The upper and lower panels follow the same format as described in panel (a).

c) Figure (c) highlights the percentage of CD8+ T-cells within the peripheral blood mononuclear population. The upper and lower panels maintain the same structure as mentioned in panel (a).

d) Figure (d) presents the percentage of TCR-β T-cells within the peripheral blood mononuclear population.

e) Figure (e) showcases the percentage of TCR gamma/delta T-cells within the peripheral blood mononuclear population.

Supplementary Figure 2

**Supplementary figure 2 (cont.) The loss of T-cells in patients with mild and severe COVID-19.**

Peripheral T-cells were isolated from healthy donors, as well as patients with mild or severe disease, and subjected to flow cytometry analysis of surface receptors. The following panels describe the results:

f) Figure presents the percentage of CD69-expressing T-cells within the CD4 and CD8+ peripheral T-cell populations. The upper panel focuses on CD4+ T-cells, while the lower panel depicts CD8+ T-cells.

g) Figure shows the percentage of PD-1-expressing T-cells within the CD4 and CD8+ peripheral T-cell populations. The upper panel represents CD4+ T-cells, while the lower panel represents CD8+ T-cells.

h) Figure displays the percentage of Notch1-expressing T-cells within the CD4 and CD8+ peripheral T-cell populations. The upper panel pertains to CD4+ T-cells, while the lower panel pertains to CD8+ T-cells.

i) Figure highlights the percentage of GZMB-expressing CD8+ peripheral T-cells. The upper panel denotes the percentage of GZMB+ cells, while the lower panel depicts a Spearman analysis illustrating the correlation between GZMB expression and disease severity.

Supplementary Figure 2

**Supplementary figure 2j-l:**  **Increase in GZMB and IFN-**γ **in patients with mild and severe COVID-19**. Peripheral T-cells were isolated from healthy donors, as well as patients with mild or severe disease, and subjected to flow cytometry analysis of surface receptors. The following panels describe the results:

j) Figure illustrates the percentage of GZMB expression in CD8+ peripheral T-cells. The upper panel focuses on CD8+ T-cells, while the lower panel presents a Spearman analysis demonstrating a correlation between GZMB expression and disease severity (r=0.31, p=\*).

k) Figure displays the percentage of IFNγ-expressing CD8+ peripheral T-cells. The upper panel pertains to CD8+ T-cells, while the lower panel showcases a Spearman analysis indicating a strong correlation between IFNγ expression and disease severity (r=0.64, p=\*\*\*\*).

l) Figure showcases the percentage of IFNγ-expressing CD4+ peripheral T-cells. The upper panel represents CD4+ T-cells, while the lower panel exhibits a Spearman analysis revealing a correlation between IFNγ expression and disease severity (r=0.37, p=\*\*\*\*).

Supplementary Figure 2

**Supplementary figure 2m:** viSNE patterns of anti-CD8, CD4, TCF1, LEF1, CD34, PD-1, CD69, IFNgamma, GzmB, EZH2, CEACAM1, Notch1 staining of T-cells from eighteen the peripheral blood of Covid-19 patients. Nine mild patients (104, 124, 174, 178, 191, 192, 197, 224, 210) and nine severe patients (172, 177,

The patient cohort consisted of nine individuals with mild symptoms (104, 124, 174, 178, 191, 192, 197, 224, 210) and nine individuals with severe symptoms (172, 177, 186, 188, 205, 211, 218, 235, 237). These patients were admitted to the McGill University Hospital Center (MUHC) following confirmation of SARS-CoV-2 infection. They provided informed consent for participation within three days of hospitalization.

Supplementary Figure 2

**Supplementary figure 2n:** viSNE patterns of anti-CD8, CD4, CD69, PD1, Notch1, TCF1, Zeb1, Bcl2, IFNgamma, GzmB, EZH2, CEACAM1, Ki67 staining of T-cells from eighteen the peripheral blood of Covid-19 patients. Nine mild patients (104, 124, 174, 178, 191, 192, 197, 224, 210) and nine severe patients (172, 177, 186, 188, 205, 211, 218, 235, 237). The patients information were mentioned in S.Figure 5.

Supplementary Figure 3

**Supplementary figure 3: Reduced SLAM6 and TCF1 expression in T-cells from patients with severe disease**.

Left panels:

Upper: The expression of SLAM-F6 is decreased in CD8+ T-cells as disease severity increases. Lower: The expression of SLAM-F6 is decreased in CD4+ T-cells as disease severity increases.

Right panels: Representative patients from each category are depicted in conventional FACs profiles.

Upper panel: The profiles demonstrate a loss of TCF1 expression when transitioning from healthy donors (HD) and patients with mild disease to those with severe disease.

Lower panel: The profiles exhibit a loss of SLAMF6 expression when transitioning from healthy donors (HD) and patients with mild disease to those with severe disease.

Supplementary Figure 4

**Supplementary figure 4: LEF1, TCF1 and ZEB1 in CD8+ T-cells from severe patients**

a) The histogram illustrates the relative loss of Notch1+, LEF1+, Zeb1+, and TCF1+ in T-cells from severe patients. The upper panel focuses on CD8+ T-cells, while the lower panel pertains to CD4+ T-cells.

b) The histogram demonstrates the relative loss of Ki67+ and Bcl2+ in T-cells from severe patients. The upper panel represents CD8+ T-cells, while the lower panel represents CD4+ T-cells.

Supplementary Figure 5

^
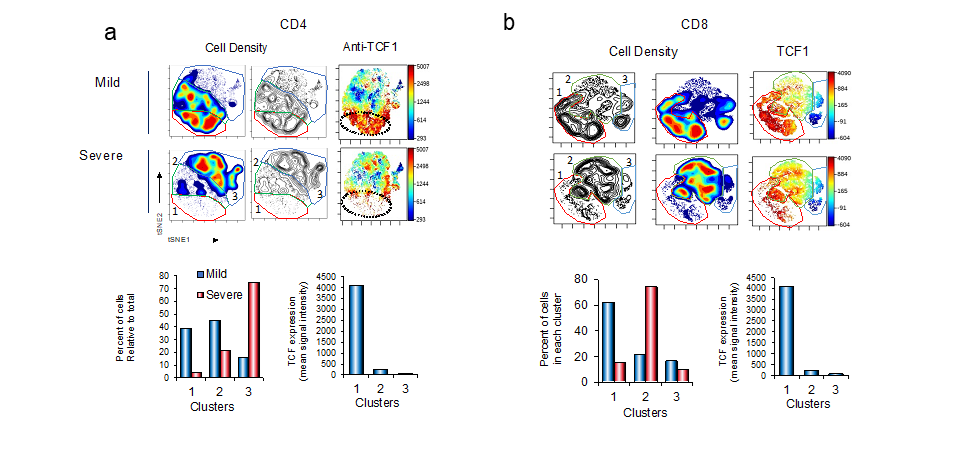
^

**Supplementary figure 5**: Additional example of viSNE profiles of other independent cohorts of mild vs severe samples showing a similar loss of TCF1 clusters in CD4 (panel a) and CD8 (panel b). Severe disease a loss of cells in CD8 and CD8 cluster 1 accompanied by the appearance of new cluster expressing with low levels of TCF1 in clusters 3. Lower histograms showed the percent of cells in each cluster and mean signal intensity/cluster.

Supplementary Figure 6

**
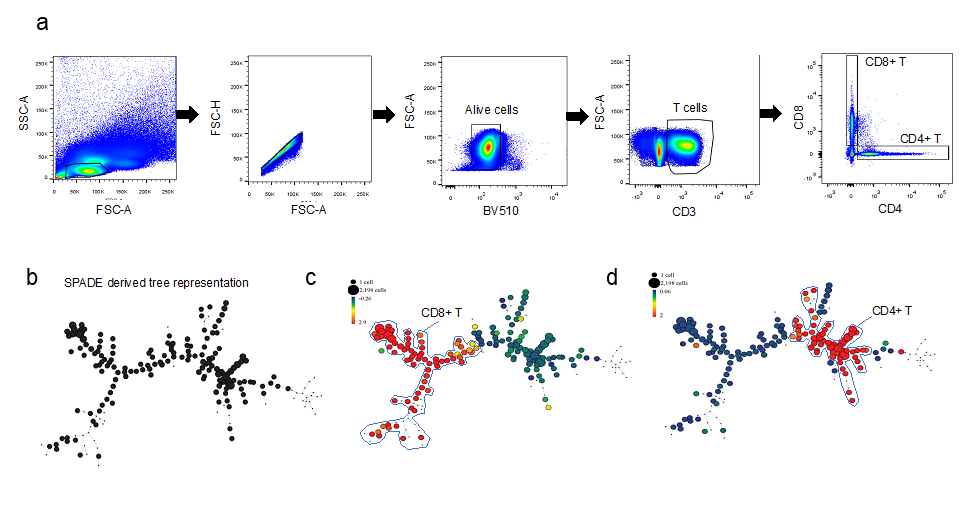
**

**Supplementary figure 6:** SPADE gating strategy.

a) The analysis involved traditional gating for CD8+ and CD4+ T cells using human peripheral blood mononuclear cells (PBMCs).

b) The SPADE profile depicted the analysis of CD3+ T cells.

c) The SPADE profile focused on CD8+ T cells within the CD3 population.

d) The SPADE profile showcased CD4+ T cells within the CD3 population. Each tree was color-coded based on the median intensity of a specific marker. The red color represented the populated regions of the tree corresponding to the cells in the respective gate.

Supplementary Figure 7

**
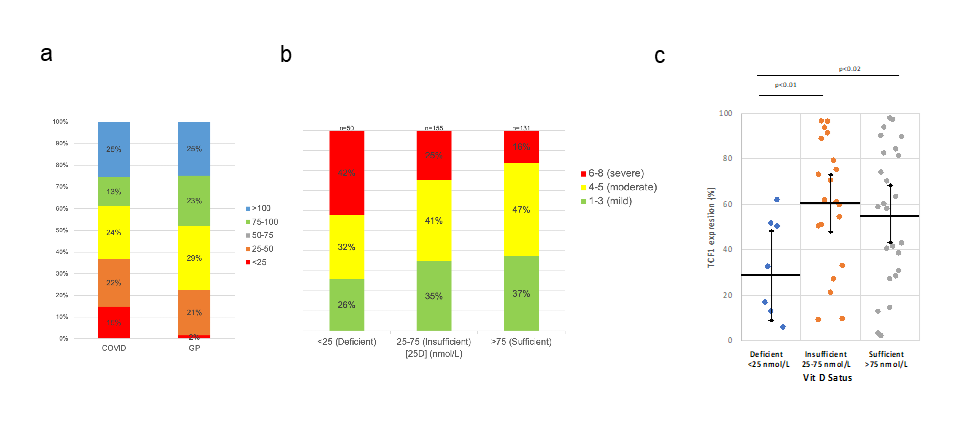
**

**Supplementary figure 7: Decline in TCF1+ T-cells in a subset of patients deficient in vitamin D.**

a) The relative rates of vitamin D status between individuals hospitalized with COVID and the general population are presented. Among 366 COVID hospitalized patients, 50 (14.9%) were found to be vitamin D deficient, whereas only 16 out of 1000 (1.6%) non-hospitalized individuals without COVID were deficient in vitamin D (p\<0.01).

b) The rates of WHO Severity scores are stratified based on vitamin D status. Among the vitamin D deficient patients, 21 out of 50 (42%) exhibited clinically severe manifestations of COVID, as indicated by their WHO severity scores, which included requirements for intubation, advanced organ support, or ultimately resulting in death. In comparison, 38 out of 155 (25%) patients with insufficient levels of vitamin D and 21 out of 131 (16%) patients with sufficient levels of vitamin D displayed severe COVID manifestations (p\<0.01).

c) The expression of TCF-1 (%TCF-1 expression) is stratified based on vitamin D status. Patients with vitamin D deficiency (n=7) exhibited lower levels of TCF-1 expression (28.5%, 95%CI 8.8-48.1%) compared to patients with vitamin D insufficiency (n=20) (60.4%, 47.2-73.6%, p\<0.01) and sufficiency (n=25) (55.6%, 43.0-68.2%, p\<0.02). It should be noted that one outlier with TCF-1 expression over 3.3 standard deviations from the mean (99.7%) was removed from the deficient group.

Supplementary Figure 8

**Supplementary figure 8: ViSNE profiles of Ki67 expression on an alternate cohort of CD4 and CD8+ T-cells.**

Left panels: For CD4+ T-cells, the transition from mild to severe disease exhibited a significant alteration in the cell distribution within clusters 1 and 2, characterized by varying levels of Ki67 expression ranging from low to moderate in mild disease. However, in the transition to severe disease, there was a pronounced loss of cells in cluster 1, accompanied by an increase in the number of cells in cluster 3, which displayed low levels of Ki67 expression. The lower histogram provides the mean fluorescence intensity (MFI) of Ki67 expression in each cluster.

Right panels: Similarly, for CD8+ T-cells, the transition from mild to severe disease involved a substantial shift in the cell distribution within cluster 1, characterized by high levels of Ki67 expression in mild disease. However, in the transition to severe disease, there was a loss of cells in cluster 1, accompanied by an increase in the number of cells in cluster 3, which exhibited low levels of Ki67 expression. The lower histogram presents the mean fluorescence intensity (MFI) of Ki67 expression in each cluster.

Supplementary Figure 9

**Supplementary figure 9:** **Examples of spanning-tree progression analysis for density-normalized events (SPADE) analysis of TCF1 and Ki67 expression in peripheral blood CD3+ T-cells from various patients** (HD: 199, 269; 36; 270; Mild: 174, 192, 197, 220 and severe: 188, 218, 286, 287). Cells were extracted, purified with Ficoll centrifugation, stained with viability stain and then with MAbs to TCF1 and Ki67. The SPADE approach identified more than 70 different cell subsets (i.e., nodes) where the size indicates cell number while the colour denotes the MFI levels of expression. SPADE profiling showed a heterogeneous expression of both markers in samples from healthy donors with high (red-orange) to moderate (light green) expression. Samples from patients with mild symptoms (WOS 3-4) showed an increase in the expression of TCF1 and Ki67 consistent with a proliferative response to the viral infection. By contrast, samples from patients with severe symptoms (WOS 5-7) showed a marked change with a reduction in TCF1 expression in most cells and an even further loss of proliferation as shown by the reduction in Ki67 expression (dark blue-black).

Supplementary Figure 10


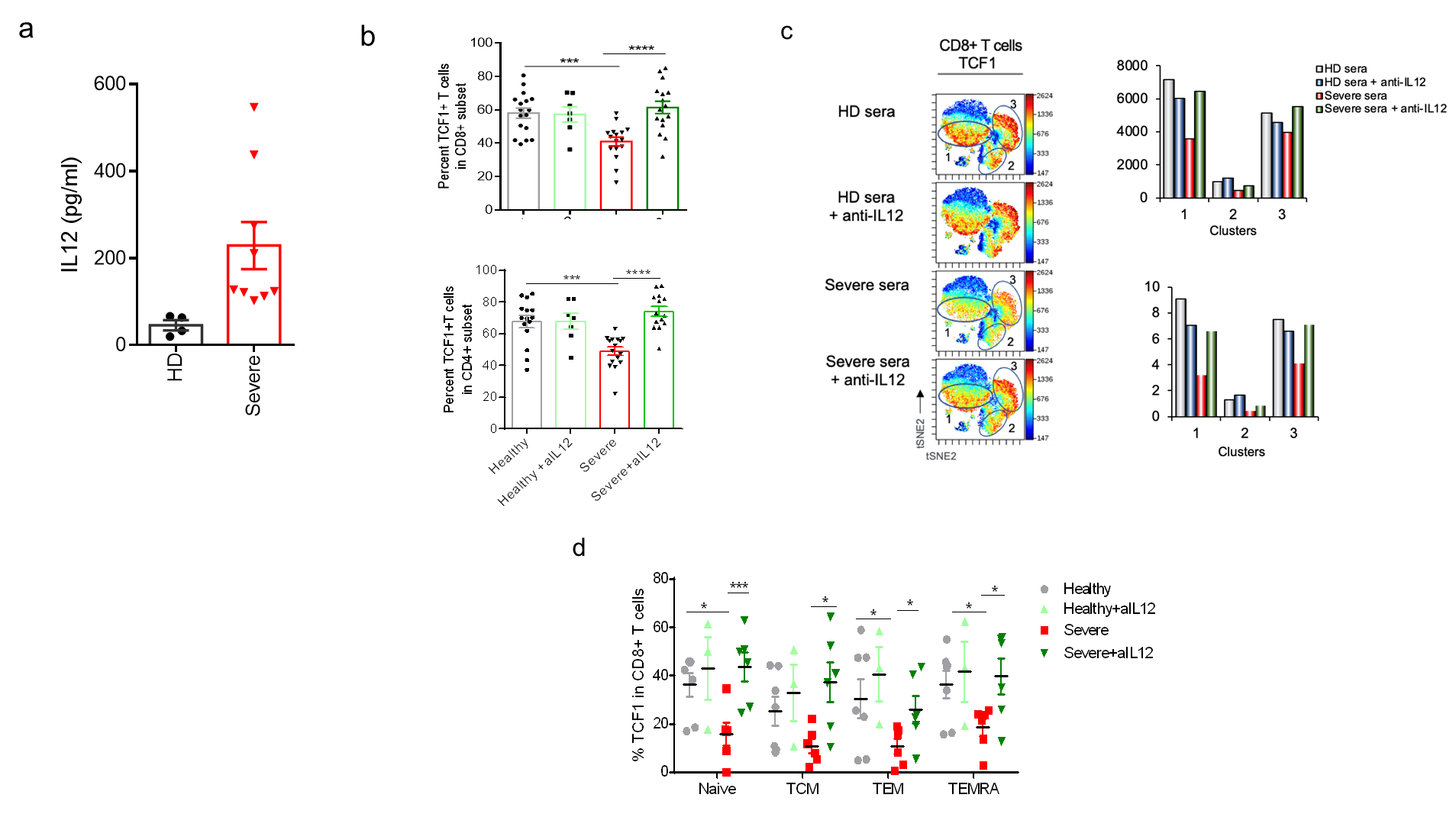


**Supplementary figure 10: Sera from severe patients suppresses the expression of TCF1 in an IL-2 dependent manner.**

a) Serum samples from healthy donors or severe patients were added to normal peripheral blood T-cells and incubated for several days, following the described methods. The percentage of TCF1+ T-cells in the CD8+ subsets exhibited reduced TCF1 expression, which was reversed when co-incubated with anti-IL12.

b) Analysis of the CD4 and CD8+ subsets revealed a decrease in the percentage of TCF1+ T-cells with reduced TCF1 expression. However, this reduction was restored by co-incubation with blocking anti-IL12. The upper panel displayed the histogram depicting the percentage of CD8+ TCF1+ T-cells, while the lower panel showed the percentage of CD4+ TCF1+ T-cells.

c) ViSNE patterns exhibited the loss of TCF1 expression in T-cells due to incubation with severe sera (top and second from the bottom), indicated by clusters 1, 2, and 3. However, when co-incubated with anti-IL12, TCF1 expression was restored, as shown in the right histograms.

d) Incubation with severe sera resulted in reduced TCF1 expression across all subsets of T-cells, including naïve, TCM, TEM, and TEMRA. However, this reduction was restored by co-incubation with IL-12.
