# Supplementary material for: Decreased progenitor TCF1 + T-cells correlate with COVID-19 disease severity
Source: Commun Biol. 2024 May 3;7:526. doi: 10.1038/s42003-024-05922-2 (PMC11068881; doi:10.1038/s42003-024-05922-2)
Supplement: Supplementary file 2 — Description of Additional Supplementary Files [file 42003_2024_5922_MOESM2_ESM.pdf]

Description of Additional Supplementary Files

File name: Supplementary Data
Description: The underlying source data for this study.
