# Supplementary material for: Decreased progenitor TCF1 + T-cells correlate with COVID-19 disease severity
Source: Commun Biol. 2024 May 3;7:526. doi: 10.1038/s42003-024-05922-2 (PMC11068881; doi:10.1038/s42003-024-05922-2)
Supplement: Supplementary file 4 — Reporting Summary [file 42003_2024_5922_MOESM4_ESM.pdf]

nature portfolio

Corresponding author(s): Christopher E Rudd

Last updated by author(s): Feb 1, 2024

Reporting Summary

Nature Portfolio wishes to improve the reproducibility of the work that we publish. This form provides structure for consistency and transparency in reporting. For further information on Nature Portfolio policies, see our Editorial Policies and the Editorial Policy Checklist.

Statistics

For all statistical analyses, confirm that the following items are present in the figure legend, table legend, main text, or Methods section.

n/a	Confirmed	
☐	☒	The exact sample size (*n*) for each experimental group/condition, given as a discrete number and unit of measurement
☐	☒	A statement on whether measurements were taken from distinct samples or whether the same sample was measured repeatedly
☐	☒	The statistical test(s) used AND whether they are one- or two-sided<br>*Only common tests should be described solely by name; describe more complex techniques in the Methods section.*
☒	☐	A description of all covariates tested
☒	☐	A description of any assumptions or corrections, such as tests of normality and adjustment for multiple comparisons
☐	☒	A full description of the statistical parameters including central tendency (e.g. means) or other basic estimates (e.g. regression coefficient) AND variation (e.g. standard deviation) or associated estimates of uncertainty (e.g. confidence intervals)
☐	☒	For null hypothesis testing, the test statistic (e.g. *F*, *t*, *r*) with confidence intervals, effect sizes, degrees of freedom and *P* value noted<br>*Give P values as exact values whenever suitable.*
☒	☐	For Bayesian analysis, information on the choice of priors and Markov chain Monte Carlo settings
☒	☐	For hierarchical and complex designs, identification of the appropriate level for tests and full reporting of outcomes
☐	☒	Estimates of effect sizes (e.g. Cohen's *d*, Pearson's *r*), indicating how they were calculated

Our web collection on statistics for biologists contains articles on many of the points above.

Software and code

Policy information about availability of computer code

Data collection	The acquisition of stained cells was carried out using a BD LSRFortessa X-20 flow cytometer and DIVA software (Beckton Dickinson).
Data analysis	1. Flow cytometry data were anslyzed by FlowJo version 10.5.3<br>2. viSNE and SPADE analyses were performed on Cytobank (https://cytobank.org).<br>3.Statistical analyses were performed using GraphPad Prism software 7.0 for window (GraphPad Software).

For manuscripts utilizing custom algorithms or software that are central to the research but not yet described in published literature, software must be made available to editors and reviewers. We strongly encourage code deposition in a community repository (e.g. GitHub). See the Nature Portfolio guidelines for submitting code & software for further information.

Data

Policy information about availability of data

All manuscripts must include a data availability statement. This statement should provide the following information, where applicable:

- Accession codes, unique identifiers, or web links for publicly available datasets
- A description of any restrictions on data availability
- For clinical datasets or third party data, please ensure that the statement adheres to our policy

Authors can confirm that all relevant data are included in the paper and its supplementary files.

Research involving human participants, their data, or biological material

Policy information about studies with human participants or human data. See also policy information about sex, gender (identity/presentation), and sexual orientation and race, ethnicity and racism.

Reporting on sex and gender	The different in sex or gender in human samples (PBMCs) does not cause any different results.
Reporting on race, ethnicity, or other socially relevant groupings	The severity of COVID-19 manifestations and clinical outcomes were assessed using the World Health Organisation (WHO)'s COVID ordinal scale. Scores of <=4 were categorized as mild manifestations, while scores >=5 were considered severe. Clinical laboratory data were collected from time points that were closest to the research blood collection, as well as time points associated with extreme values.
Population characteristics	Please see the supplementary table S1 and S2.
Recruitment	This study was conducted in accordance with the ethical guidelines set by the McGill University Health Centre Research Institute (MUHC-RI) Ethics Board, under the approved study protocol (#2021-6081). Informed consent was obtained from patients admitted to the McGill University Health Centre (MUHC) with confirmed SARS-CoV-2 infection between April 2020 and March 2021. Additional peripheral blood samples from uninfected healthy adults were obtained from the Hema-Quebec blood bank, following ethical approval by the CR-HMR Ethical Approval (Le Comité de protection des animaux du CIUSSS de l'Est-de-l'Île-de-Montréal [CPA-CEMTL], F06 CPA-21061 du projet 2017-1346, 2017-JA-001).
Ethics oversight	by the McGill University Health Centre Research Institute (MUHC-RI) Ethics Board, under the approved study protocol (#2021-6081) and the CR-HMR Ethical Approval (Le Comité de protection des animaux du CIUSSS de l'Est-de-l'Île-de-Montréal [CPA-CEMTL], F06 CPA-21061 du projet 2017-1346, 2017-JA-001).

Note that full information on the approval of the study protocol must also be provided in the manuscript.

Field-specific reporting

Please select the one below that is the best fit for your research. If you are not sure, read the appropriate sections before making your selection.

☒ Life sciences ☐ Behavioural & social sciences ☐ Ecological, evolutionary & environmental sciences

For a reference copy of the document with all sections, see nature.com/documents/nr-reporting-summary-flat.pdf

Life sciences study design

All studies must disclose on these points even when the disclosure is negative.

Sample size	The sample size were mentioned in Methods part and Figure legend. In total: Healthy Donors (HD) (n=27), Mild cases (n=56), and Severe cases (n=38).
Data exclusions	No data was excluded.
Replication	The samples were provided enough for examination.
Randomization	Human PBMCs samples were taken randomly from COVID-19 patients or from healthy donors.
Blinding	The COVID-19 samples severity were defined base on the WHO score. All samples must be named correctly and transfered in special condition.

Reporting for specific materials, systems and methods

We require information from authors about some types of materials, experimental systems and methods used in many studies. Here, indicate whether each material, system or method listed is relevant to your study. If you are not sure if a list item applies to your research, read the appropriate section before selecting a response.

Materials & experimental systems

n/a	Involved in the study
☐	☒ Antibodies
☒	☐ Eukaryotic cell lines
☒	☐ Palaeontology and archaeology
☒	☐ Animals and other organisms
☒	☐ Clinical data
☒	☐ Dual use research of concern
☒	☐ Plants

Methods

n/a	Involved in the study
☐	☐ ChIP-seq
☐	☒ Flow cytometry
☐	☐ MRI-based neuroimaging

Antibodies

Antibodies used

AF700-conjugated anti-CD3 (clone: UCHT1), Cat. 561027-BD
APC-conjugated anti-TCR alpha/beta (clone: IP26), Cat. 17-9986-42-eBioscence
FITC-conjugated anti-TCR gamma/delta (clone: B1), Cat. 331208-Biolegend
BV785-conjugated anti-CD8 (clone: RPA-T8), Cat. 301046-Biolegend
BUV395-conjugated anti-CD4 (clone: RPA-T4), Cat. 564724-BD
BV605-conjugated anti-PD-1 (clone: EH12.1), Cat. 563245-BD
APC-Cy7-conjugated anti-CD69 (clone: FN50), Cat. 560912-BD
BV650-conjugated anti-Notch (clone: MHN1-519), Cat. 743909-BD
PE-conjugated anti-TCF1 (clone: 7F11A10), Cat. 655208-Biolegend
PEcy7-conjugated anti-IFNg (clone: 4S.B3), Cat. 557844-BD
PE-CF594-conjugated anti-Granzyme B (clone: GB11), Cat. 562462-BD
FITC-conjugated anti-Ki67 (clone: 11F6), Cat. 151204-Biolegend
BV421-conjugated anti-Bcl2 (clone: 100), Cat. 658709-Biolegend
FITC active Caspase-3 apoptosis kit, Cat, 550480 - BD

Validation

Alexa Fluor® 700 Mouse Anti-Human CD3, Reactive: Human, Application: Flow cytometry
TCR alpha/beta Monoclonal Antibody (IP26) APC, eBioscience, Reactive: Human, Application: Flow cytometry
FITC anti-human TCR γ/δ Antibody, Reactive: Human, Cynomolgus, Rhesus; Application: Flow cytometry
Brilliant Violet 785™ anti-human CD8a Antibody, Reactive: Human, Cynomolgus, Rhesus; Application: Flow cytometry
BUV395 Mouse Anti-Human CD4, Reactive: Human, Application: Flow cytometry
BV605 Mouse Anti-Human CD279 (PD-1), Reactive: Human, Application: Flow cytometry
APC-Cy™7 Mouse Anti-Human CD69, Reactive: Human, Rhesus, Cynomolgus, Baboon; Application: Flow cytometry
BD OptiBuild™ BV650 Mouse Anti-Human Notch1, Reactive: Human, Application: Flow cytometry
PE anti-TCF1 (TCF7) Antibody (clone: 7F11A10), Reactive: Human, Application: Flow cytometry
PE-Cy™7 Mouse Anti-Human IFN-γ, Reactive: Human, Rhesus, Cynomolgus, Baboon; Application: Flow cytometry
PE-CF594 Mouse Anti-Human Granzyme B, Reactive: Human, Application: Flow cytometry
Alexa Fluor® 488 anti-mouse/human Ki-67 Antibody, Reactive:Mouse, Human; Application: Flow cytometry, ICC, IHC-F
Brilliant Violet 421™ anti-Bcl-2 Antibody, Reactive: Human, Application: Flow cytometry
BD Pharmingen™ FITC Active Caspase-3 Apoptosis Kit, Human, Application: Flow cytometry

Plants

Seed stocks

Report on the source of all seed stocks or other plant material used. If applicable, state the seed stock centre and catalogue number. If plant specimens were collected from the field, describe the collection location, date and sampling procedures.

Novel plant genotypes

Describe the methods by which all novel plant genotypes were produced. This includes those generated by transgenic approaches, gene editing, chemical/radiation-based mutagenesis and hybridization. For transgenic lines, describe the transformation method, the number of independent lines analyzed and the generation upon which experiments were performed. For gene-edited lines, describe the editor used, the endogenous sequence targeted for editing, the targeting guide RNA sequence (if applicable) and how the editor was applied.

Authentication

Describe any authentication procedures for each seed stock used or novel genotype generated. Describe any experiments used to assess the effect of a mutation and, where applicable, how potential secondary effects (e.g. second site T-DNA insertions, mosiacism, off-target gene editing) were examined.

ChIP-seq

Data deposition

☐ Confirm that both raw and final processed data have been deposited in a public database such as GEO.

☐ Confirm that you have deposited or provided access to graph files (e.g. BED files) for the called peaks.

Data access links
May remain private before publication.

For "Initial submission" or "Revised version" documents, provide reviewer access links. For your "Final submission" document, provide a link to the deposited data.

Files in database submission | *Provide a list of all files available in the database submission.*

Genome browser session (e.g. UCSC) | *Provide a link to an anonymized genome browser session for "Initial submission" and "Revised version" documents only, to enable peer review. Write "no longer applicable" for "Final submission" documents.*

Methodology

Replicates | *Describe the experimental replicates, specifying number, type and replicate agreement.*

Sequencing depth | *Describe the sequencing depth for each experiment, providing the total number of reads, uniquely mapped reads, length of reads and whether they were paired- or single-end.*

Antibodies | *Describe the antibodies used for the ChIP-seq experiments; as applicable, provide supplier name, catalog number, clone name, and lot number.*

Peak calling parameters | *Specify the command line program and parameters used for read mapping and peak calling, including the ChIP, control and index files used.*

Data quality | *Describe the methods used to ensure data quality in full detail, including how many peaks are at FDR 5% and above 5-fold enrichment.*

Software | *Describe the software used to collect and analyze the ChIP-seq data. For custom code that has been deposited into a community repository, provide accession details.*

Flow Cytometry

Plots

Confirm that:

☒ The axis labels state the marker and fluorochrome used (e.g. CD4-FITC).

☒ The axis scales are clearly visible. Include numbers along axes only for bottom left plot of group (a 'group' is an analysis of identical markers).

☒ All plots are contour plots with outliers or pseudocolor plots.

☒ A numerical value for number of cells or percentage (with statistics) is provided.

Methodology

Sample preparation | To isolate peripheral blood mononuclear cells (PBMCs), the density gradient centrifugation method was employed at the MUHC-RI. Peripheral blood was collected into K2 EDTA tubes (BD) and carefully layered above an appropriate volume of density gradient medium in 15ml tubes (e.g., Lymphoprep by Stemcell Technologies). The tubes were centrifuged at room temperature (RT) for 30 minutes at 400g. Subsequently, the plasma fraction was collected and stored. The PBMC layer, located between the plasma and the density gradient medium, was carefully collected and washed twice with phosphate-buffered saline (PBS). The isolated PBMCs were then stained for viability, counted, and fixed in 2% paraformaldehyde at RT for 20 minutes to inactivate the SARS coronavirus. After fixation, the PBMCs were washed twice with fluorescence-activated cell sorting (FACS) buffer (PBS containing 2% fetal bovine serum), centrifuged (400g, 5 min, RT), and finally frozen in freezing media (90% FBS/10% DMSO) at -80°C until further FACS staining.

Instrument | The acquisition of stained cells was carried out using a BD LSRFortessa X-20 flow cytometer and DIVA software (Beckton Dickinson)

Software | Flow cytometry data were anslyzed by FlowJo version 10.5.3

Cell population abundance | *Describe the abundance of the relevant cell populations within post-sort fractions, providing details on the purity of the samples and how it was determined.*

Gating strategy |
Gating FSC-A/SSC-A: for indicate lymphocyte;
Gating FSC-A/FSC-H: for indicate singlet,
Gating FSC-A/BV510: for indicate alive cells;
Gating FSC-A/CD3: for indicate T cells,
Gating CD8/CD4: for indicate CD8+ and CD4+ T cells;
Continuously gating for intracellular staining marker in each population CD8+ or CD4+ T cells.

☒ Tick this box to confirm that a figure exemplifying the gating strategy is provided in the Supplementary Information.

Magnetic resonance imaging

Experimental design

Design type | *Indicate task or resting state; event-related or block design.*

Design specifications | *Specify the number of blocks, trials or experimental units per session and/or subject, and specify the length of each trial or block (if trials are blocked) and interval between trials.*

Behavioral performance measures | *State number and/or type of variables recorded (e.g. correct button press, response time) and what statistics were used to establish that the subjects were performing the task as expected (e.g. mean, range, and/or standard deviation across subjects).*

Acquisition

Imaging type(s) | *Specify: functional, structural, diffusion, perfusion.*

Field strength | *Specify in Tesla*

Sequence & imaging parameters | *Specify the pulse sequence type (gradient echo, spin echo, etc.), imaging type (EPI, spiral, etc.), field of view, matrix size, slice thickness, orientation and TE/TR/flip angle.*

Area of acquisition | *State whether a whole brain scan was used OR define the area of acquisition, describing how the region was determined.*

Diffusion MRI ☐ Used ☐ Not used

Preprocessing

Preprocessing software | *Provide detail on software version and revision number and on specific parameters (model/functions, brain extraction, segmentation, smoothing kernel size, etc.).*

Normalization | *If data were normalized/standardized, describe the approach(es): specify linear or non-linear and define image types used for transformation OR indicate that data were not normalized and explain rationale for lack of normalization.*

Normalization template | *Describe the template used for normalization/transformation, specifying subject space or group standardized space (e.g. original Talairach, MNI305, ICBM152) OR indicate that the data were not normalized.*

Noise and artifact removal | *Describe your procedure(s) for artifact and structured noise removal, specifying motion parameters, tissue signals and physiological signals (heart rate, respiration).*

Volume censoring | *Define your software and/or method and criteria for volume censoring, and state the extent of such censoring.*

Statistical modeling & inference

Model type and settings | *Specify type (mass univariate, multivariate, RSA, predictive, etc.) and describe essential details of the model at the first and second levels (e.g. fixed, random or mixed effects; drift or auto-correlation).*

Effect(s) tested | *Define precise effect in terms of the task or stimulus conditions instead of psychological concepts and indicate whether ANOVA or factorial designs were used.*

Specify type of analysis: ☐ Whole brain ☐ ROI-based ☐ Both

Statistic type for inference | *Specify voxel-wise or cluster-wise and report all relevant parameters for cluster-wise methods.*

(See Eklund et al. 2016)

Correction | *Describe the type of correction and how it is obtained for multiple comparisons (e.g. FWE, FDR, permutation or Monte Carlo).*

Models & analysis

n/a	Involved in the study
☒	☐ Functional and/or effective connectivity
☐	☒ Graph analysis
☒	☐ Multivariate modeling or predictive analysis

Graph analysis | All data are expressed as mean ± SEM. A t-test was used when only two groups were compared, and a one-way ANOVA was used when more than two groups were compared. A two-way ANOVA was used for multiple comparison procedures that involved two independent variables. A difference in mean values between groups was significant when $p < 0.05$ *; $p < 0.01$ ** and $p < 0.001$ ***.
